# Collective All‐Carbon Magnetism in Triangulene Dimers†

**DOI:** 10.1002/anie.202002687

**Published:** 2020-05-18

**Authors:** Shantanu Mishra, Doreen Beyer, Kristjan Eimre, Ricardo Ortiz, Joaquín Fernández‐Rossier, Reinhard Berger, Oliver Gröning, Carlo A. Pignedoli, Roman Fasel, Xinliang Feng, Pascal Ruffieux

**Affiliations:** ^1^ nanotech@surfaces Laboratory Empa-Swiss Federal Laboratories for Materials Science and Technology 8600 Dübendorf Switzerland; ^2^ Center for Advancing Electronics and Department of Chemistry and Food Chemistry Technical University of Dresden 01062 Dresden Germany; ^3^ Department of Applied Physics University of Alicante 03690 Sant Vicent del Raspeig Spain; ^4^ Department of Chemical Physics University of Alicante 03690 Sant Vicent del Raspeig Spain; ^5^ QuantaLab International Iberian Nanotechnology Laboratory 4715-330 Braga Portugal; ^6^ Department of Chemistry and Biochemistry University of Bern 3012 Bern Switzerland

**Keywords:** magnetism, nanographenes, on-surface synthesis, scanning probe microscopy, surface chemistry

## Abstract

Triangular zigzag nanographenes, such as triangulene and its π‐extended homologues, have received widespread attention as organic nanomagnets for molecular spintronics, and may serve as building blocks for high‐spin networks with long‐range magnetic order, which are of immense fundamental and technological relevance. As a first step towards these lines, we present the on‐surface synthesis and a proof‐of‐principle experimental study of magnetism in covalently bonded triangulene dimers. On‐surface reactions of rationally designed precursor molecules on Au(111) lead to the selective formation of triangulene dimers in which the triangulene units are either directly connected through their minority sublattice atoms, or are separated via a 1,4‐phenylene spacer. The chemical structures of the dimers have been characterized by bond‐resolved scanning tunneling microscopy. Scanning tunneling spectroscopy and inelastic electron tunneling spectroscopy measurements reveal collective singlet–triplet spin excitations in the dimers, demonstrating efficient intertriangulene magnetic coupling.

## Graphical abstract

**The on‐surface synthesis** of covalently bonded triangulene dimers with or without a 1,4‐phenylene spacer was achieved on Au(111). Scanning tunneling spectroscopy measurements revealed collective magnetism in the dimers in the form of singlet–triplet spin excitations, demonstrating efficient and tunable intertriangulene magnetic coupling.

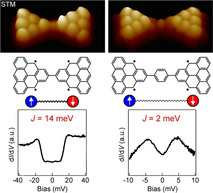

## Introduction

**Figure 1:**
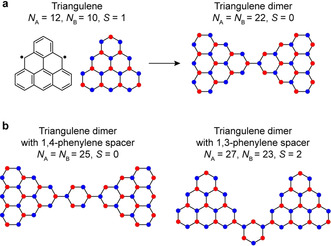
Tunability of magnetic coupling in triangulene dimers. a) Chemical structure of triangulene with the carbon atoms of the two interpenetrating triangular sublattices highlighted as blue and red filled circles (left). *N*_A_ and *N*_B_ denote the number of carbon atoms in the *A* and *B* sublattices, respectively. Triangulene exhibits a sublattice imbalance of two, with the majority sublattice atoms located at the zigzag edges. Direct coupling of two triangulene units through their minority sublattice atoms leads to no sublattice imbalance in the dimer (right), leading to a low‐spin ground state. b) Schematic showing triangulene dimers with 1,4‐phenylene (left) and 1,3‐phenylene (right) spacers. The dimer with the 1,3‐phenylene spacer contains a net sublattice imbalance of four in the structure, leading to a high‐spin ground state.

The fusion of benzenoid rings in a triangular fashion leads to the generation of triangular zigzag nanographenes (TZNGs) for which no Kekulé valence structures can be drawn without leaving unpaired electrons.^1^ The underlying basis for the non‐Kekulé structure of TZNGs is an inherent sublattice imbalance in the bipartite honeycomb lattice such that the simultaneous pairing of all p_*z*_‐electrons into π‐bonds is impossible (Figure 1 a).^2^, ^3^, ^4^ Application of Ovchinnikov's rule^5^, ^6^ predicts an increasing ground‐state total spin quantum number *S* with increasing size of the TZNGs. Derivatives of phenalenyl radical^7^ (three fused rings, *S*=1/2) and triangulene^8^, ^9^ (six fused rings, *S*=1) have been obtained in solution, and their magnetic ground states have been confirmed by electron paramagnetic resonance spectroscopy. In the last three years, unsubstituted triangulene^10^ and its larger homologues,^11^, ^12^ that is, π‐extended [4]‐ and [5]‐triangulene containing ten and fifteen fused rings, with *S*=3/2 and 2, respectively, have been obtained on metal and insulator surfaces, and their electronic structures have been elucidated at submolecular resolution using scanning tunneling microscopy and spectroscopy (STM and STS). A range of applications have been envisaged for TZNGs in molecular electronics and spintronics such as spin filters,^13^, ^14^ qubits for quantum information processing,^15^ and electrically controllable magnetic switches.^16^, ^17^ Given their high‐spin ground states, interesting fundamental and technological prospects lie in the construction of one‐dimensional chains and two‐dimensional networks incorporating TZNGs as building blocks—such as the discovery of elusive quantum states of matter^18^ and room‐temperature long‐range magnetic ordering.^19^, ^20^, ^21^ With the advent of on‐surface synthesis as a chemical toolbox,^22^ fabrication of extended TZNG nanostructures seems feasible on metal surfaces, given the proper chemical precursor design. Figure 1 illustrates the versatility of TZNG nanostructures. Connecting two triangulene units directly through their minority sublattice carbon atoms does not produce a net sublattice imbalance in the structure, and is thus expected to yield an *S*=0 ground state as per Ovchinnikov's rule (Figure 1 a), which could either correspond to a magnetic, open‐shell singlet or a non‐magnetic, closed‐shell ground state. Introduction of an organic spacer in the structure serves not only to tune the magnetic coupling between the triangulene units, but also to modify the magnetic correlations, leading to high‐ or low‐spin ground states. As shown in Figure 1 b, while separation of two triangulene units by a 1,4‐phenylene spacer is expected to result in an *S*=0 ground state, separation through a 1,3‐phenylene spacer generates a net sublattice imbalance in the structure, and therefore should result in a ground state with *S*>0. Therefore, a range of nanoarchitectures based on TZNGs can be conceived with tunable coupling strengths and magnetic ground states.

**Scheme 1:**
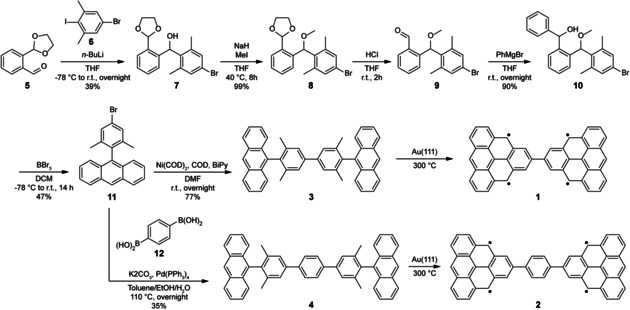
Synthetic route towards triangulene dimers reported in this work.

In this regard, two fundamental problems need to be solved. First, a direct proof of magnetism in TZNGs on metal surfaces, such as spin excitations or Kondo interactions between unpaired spins and conduction electrons of surfaces, is lacking.^23^, ^24^, ^25^, ^26^ Current experimental interpretation of magnetism in TZNGs is indirect, and relies on 1) spectroscopic detection of the spin‐split frontier molecular orbitals and 2) subsequent comparison of the experimental Coulomb gap with theoretical predictions to estimate the magnetic ground state. Second, it is imperative to demonstrate that spins in TZNG nanostructures can couple on a metal surface to result in a measurable collective magnetic ground state. Here, we devise a strategy to address the above problems through on‐surface synthesis of triangulene dimers where the constituent triangulene units are either directly connected through a carbon–carbon bond through their minority sublattice atoms (**1**), or are separated via a 1,4‐phenylene spacer (**2**). Our synthetic strategy relies on the solution synthesis of precursor molecules 9,9′‐(3,3′,5,5′‐tetramethyl‐[1,1′‐biphenyl]‐4,4′‐diyl)dianthracene (**3**) and 9,9′‐(3,3′′,5,5′′‐tetramethyl‐[1,1′:4′,1′′‐terphenyl]‐4,4′′‐diyl)dianthracene (**4**; Scheme 1, see the Supporting Information for detailed solution synthesis data), which, when annealed on a Au(111) surface, yield **1** and **2**, respectively. Using STS and STM‐based inelastic electron tunneling spectroscopy (IETS), we then unraveled unambiguous spectroscopic signatures of collective magnetism in **1** and **2** in the form of singlet–triplet spin excitations.

## Results and Discussion

To synthesize precursors **3** and **4**, the key building block 9‐(4‐bromo‐2,6‐dimethylphenyl)anthracene (**11**) was obtained by a five‐step process (Scheme 1). 2‐(1,3‐Dioxolan‐2‐yl)benzaldehyde (**5**) was reacted with 5‐bromo‐2‐iodo‐*m*‐xylene (**6**) at low temperature to form (2‐(1,3‐dioxolan‐2‐yl)phenyl(4‐bromo‐2,6‐dimethylphenyl)methanol (**7**). Because of the instability of **7**, a Williamson ether synthesis was carried out with sodium hydride (NaH) and methyl iodide (MeI). Compound **8** was then stirred in the presence of 10 % aqueous hydrochloric acid (HCl) to cleave off the acetal group and to afford benzaldehyde **9**. The crude aldehyde **9** was directly treated with an excess of phenylmagnesium bromide (PhMgBr) to afford compound **10**. Eventually, an intramolecular Friedel–Crafts‐type cyclization under Lewis acid conditions gave **11**. A subsequent nickel‐catalyzed Yamamoto coupling of **11** under glove‐box conditions resulted in the formation of the first target precursor **3** in 77 % yield. On the other hand, a Pd‐catalyzed Suzuki cross‐coupling between **11** and 1,4‐phenylenediboronic acid (**12**) afforded the second target precursor **4** in 35 % yield.

**Figure 2:**
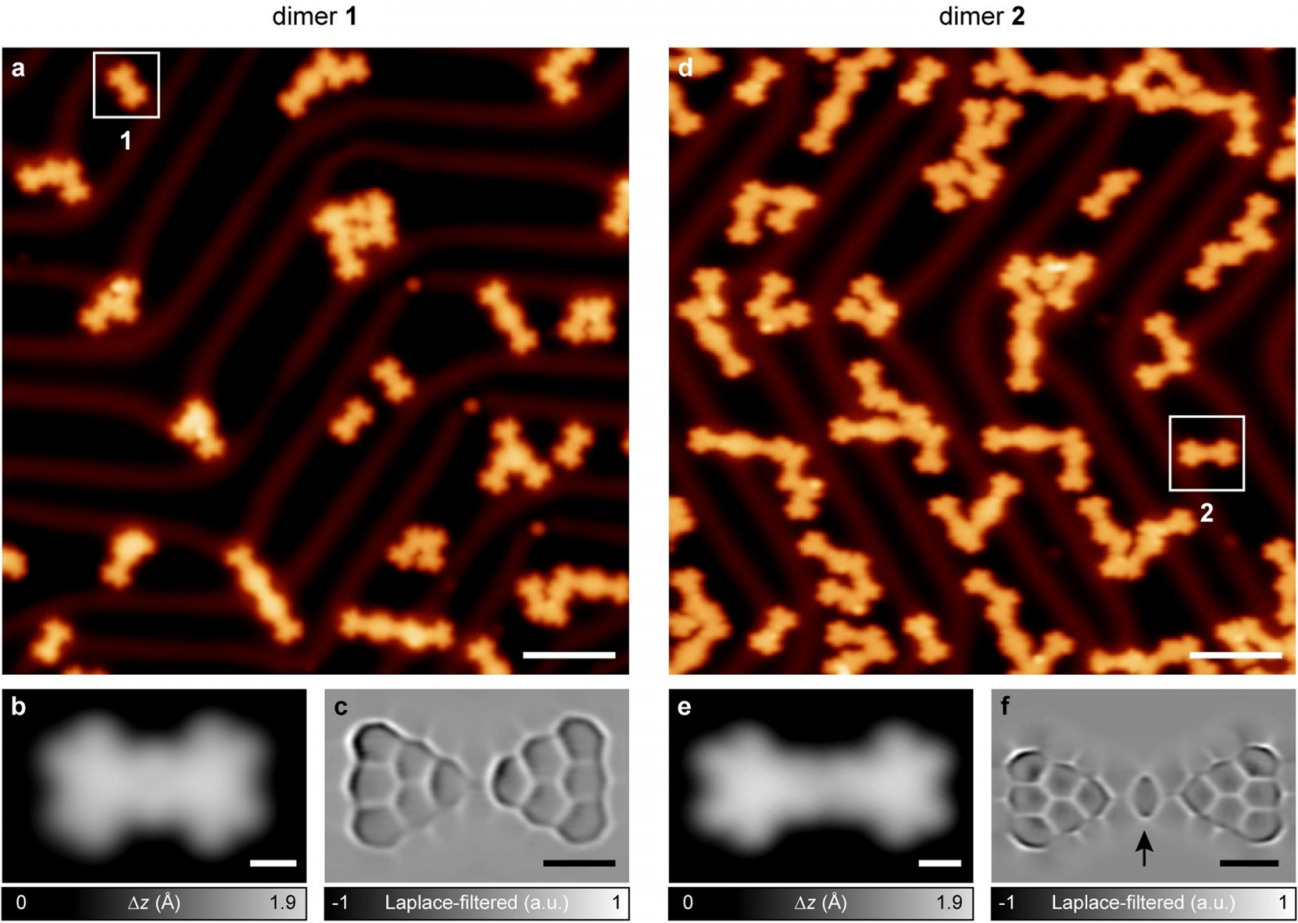
On‐surface synthesis and structural characterization of **1** and **2**. a, d) Overview STM images after annealing precursors **3** (a) and **4** (d) on Au(111) at 300 °C. Tunneling parameters: *V*=−600 mV, *I*=100 pA (a) and *V*=−600 mV, *I*=20 pA (d). Isolated **1** and **2** molecules are highlighted with squares. b, e) High‐resolution STM images of **1** (b) and **2** (e). Tunneling parameters: *V*=−600 mV, *I*=200 pA (b) and *V*=−600 mV, *I*=150 pA (e). c, f) Corresponding Laplace‐filtered ultrahigh‐resolution STM images of **1** (c) and **2** (f). The arrow in (f) highlights the 1,4‐phenylene spacer. Open feedback parameters: *V*=−5 mV, *I*=50 pA; Δ*z*=−0.8 Å (c) and −0.9 Å (f). Scale bars: 5 nm (a, d) and 0.5 nm (b, c, e, f).

Towards the synthesis of **1**, a sub‐monolayer coverage of **3** was deposited on a Au(111) surface held at room temperature, and annealed to 300 °C to promote oxidative cyclization of the methyl groups. STM imaging of the surface after the annealing step revealed isolated dumbbell‐shaped molecules and covalently bonded oligomers (Figure 2 a). Figure 2 b presents a high‐resolution STM image of an individual molecule, which shows characteristic lobed signatures in the local density of states (LDOS). We conducted ultrahigh‐resolution STM imaging with a carbon monoxide functionalized tip^27^, ^28^ to obtain the bond‐resolved structure of the molecule, which confirmed the successful formation of **1** (Figure 2 c and Supporting Information, Figure S1). The synthesis of **2** was conducted in a similar manner. STM imaging after a 300 °C annealing step of a Au(111) surface with pre‐deposited **4** revealed isolated molecules similar in appearance to **1** (Figures 2 d, e), and ultrahigh‐resolution STM imaging confirmed the successful formation of **2** (Figure 2 f).

**Figure 3:**
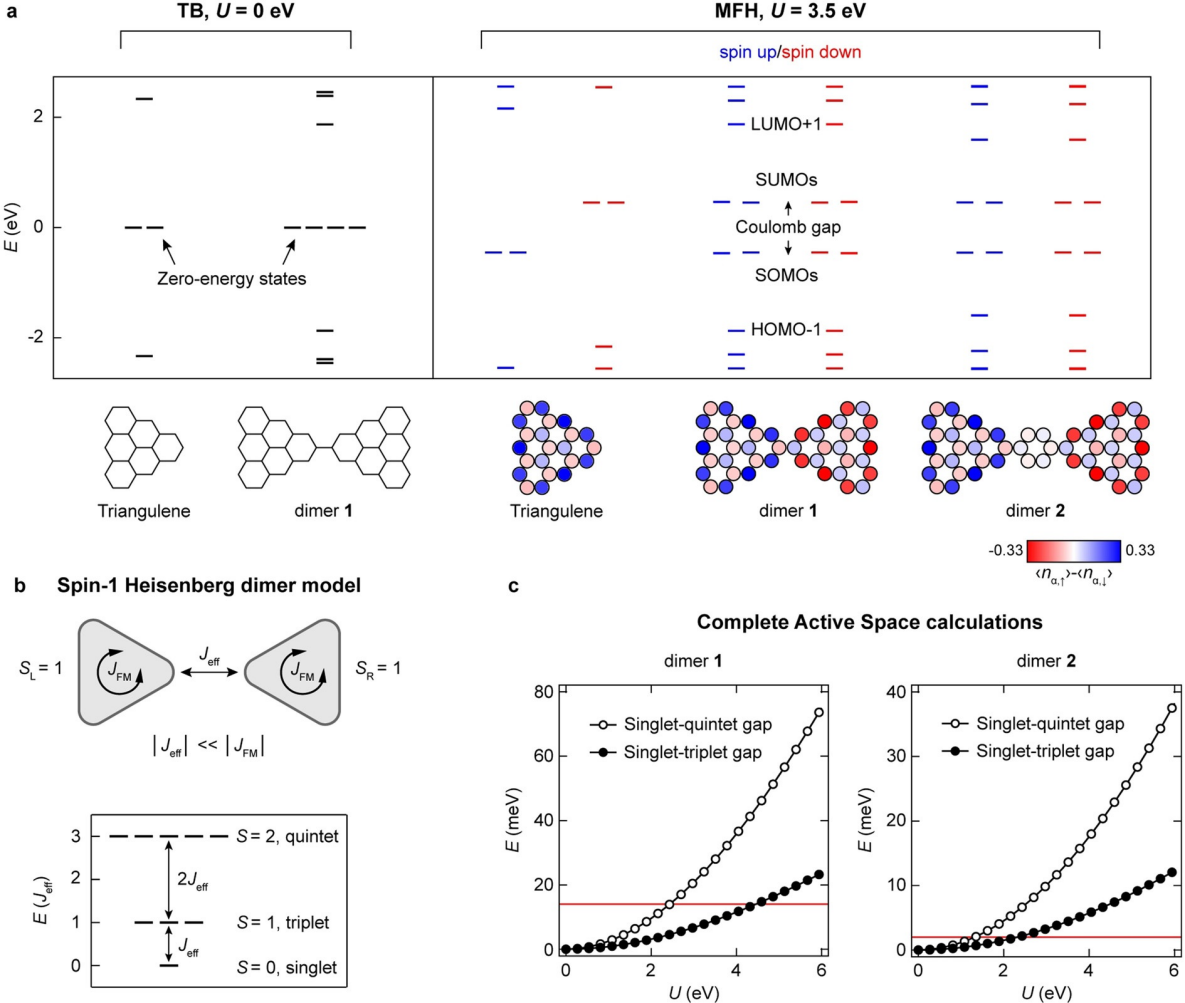
Theoretical electronic and magnetic characterization of **1** and **2**. a) Nearest‐neighbor TB energy spectra of triangulene and **1** (left) and MFH energy spectra of triangulene, **1**, and **2** along with the corresponding spin polarization plots (right). *U* denotes the on‐site Coulomb repulsion. b) Schematic illustration of the spin‐1 Heisenberg dimer model for **1** and **2** (top), with the corresponding energy level scheme from an analytical solution of the Heisenberg dimer model for an antiferromagnetic coupling between the triangulene units (bottom). *S*_L/R_ denotes the total spin quantum numbers of the left/right triangulene units. c) Energies of the open‐shell triplet and quintet states of **1** and **2** with respect to their open‐shell singlet ground states calculated in the CAS(6,6) approximation, and plotted as a function of *U*. The red solid lines indicate the experimental singlet–triplet gaps of 14 meV and 2 meV for **1** and **2**, respectively.

Figure 3 shows the electronic and magnetic structures of triangulene and the dimers **1** and **2** at successively more refined levels of theory. We started by analyzing the three systems in the nearest‐neighbor tight binding (TB) model, which disregards any electron–electron interaction. The salient features in the TB energy spectra correspond to two and four non‐bonding zero‐energy states (ZESs) for triangulene^2^, ^29^ and **1** (and **2**, not shown), respectively (Figure 3 a). The ZESs of individual triangulene units survive in the dimers **1** and **2** given that the bridging carbon–carbon bond of **1** (and the benzenoid ring of **2**) connects minority sublattice sites of the triangulene units where the ZESs have zero amplitude (Figure 1). Inclusion of electron–electron correlations within the mean‐field Hubbard (MFH) model lifts the degeneracy of the ZESs in **1** and **2**, leading to the formation of singly occupied and singly unoccupied molecular orbitals (SOMOs and SUMOs), along with the opening up of a sizeable Coulomb gap (Figure 3 a). The lowest‐energy MFH solution corresponds to an antiferromagnetic order between the triangulene units of **1** and **2**, leading to an *S=*0 open‐shell singlet ground state, in agreement with Ovchinnikov's rule. In the case of a single triangulene molecule, the magnetic ground state has been found to be an open‐shell triplet (*S=*1), which is approximately 500 meV lower in energy than the closed‐shell first excited state.^30^ Accordingly, **1** and **2** may be considered as weakly coupled Heisenberg spin‐1 dimers as the effective exchange coupling *between* the triangulene units, *J*_eff_, can be assumed to be much smaller than the strong ferromagnetic coupling *within* the triangulene units, *J*_FM_<0 (Figure 3 b). Analytical solution of the Heisenberg dimer model (Supporting Information, Note S1) for an antiferromagnetic coupling *J*_eff_>0 predicts an open‐shell singlet ground state, with the open‐shell triplet state at energy *J*_eff_ as the first and the open‐shell quintet (*S*=2) state at energy 3 *J*_eff_ as the second excited state, as shown in Figure 3 b. To obtain quantitative values of *J*_eff_, we solved the Hubbard model for **1** and **2** using the exact diagonalization in the complete active space (CAS) formed by six electrons in six single particle states—that is, the four non‐bonding states, along with the HOMO‐1 and LUMO+1 states, where HOMO and LUMO refer to the highest occupied and the lowest unoccupied molecular orbitals, respectively (see the Supporting Information for method details). The Hubbard model is known to give results in line with those of advanced quantum chemistry methods.^30^ The exact diagonalization of CAS(6,6) for both **1** and **2** yielded an open‐shell singlet ground state, followed by the open‐shell triplet and quintet states as the first and second excited states, respectively. The energies of the states are related as *E*(*S*=2)−*E*(*S*=0)=3 [*E*(*S*=1)−*E*(*S*=0)] for both **1** and **2**, thus conforming to the effective Heisenberg dimer model of two antiferromagnetically coupled spin‐1 systems. The magnetic excitation spectra of **1** and **2** calculated in the CAS(6,6) model, which approximate the singlet–triplet and singlet–quintet gaps, are shown in Figure 3 c as a function of the on‐site Coulomb repulsion *U*. Our calculations show that at a given *U*, the excitation energies are much larger for **1** than for **2**. Furthermore, the *J*_eff_ values for both **1** and **2** are at least thirty times smaller than the intratriangulene exchange coupling *J*_FM_, confirming the basic assumption |*J*_eff_|≪|*J*_FM_| underlying the Heisenberg dimer model.

**Figure 4:**
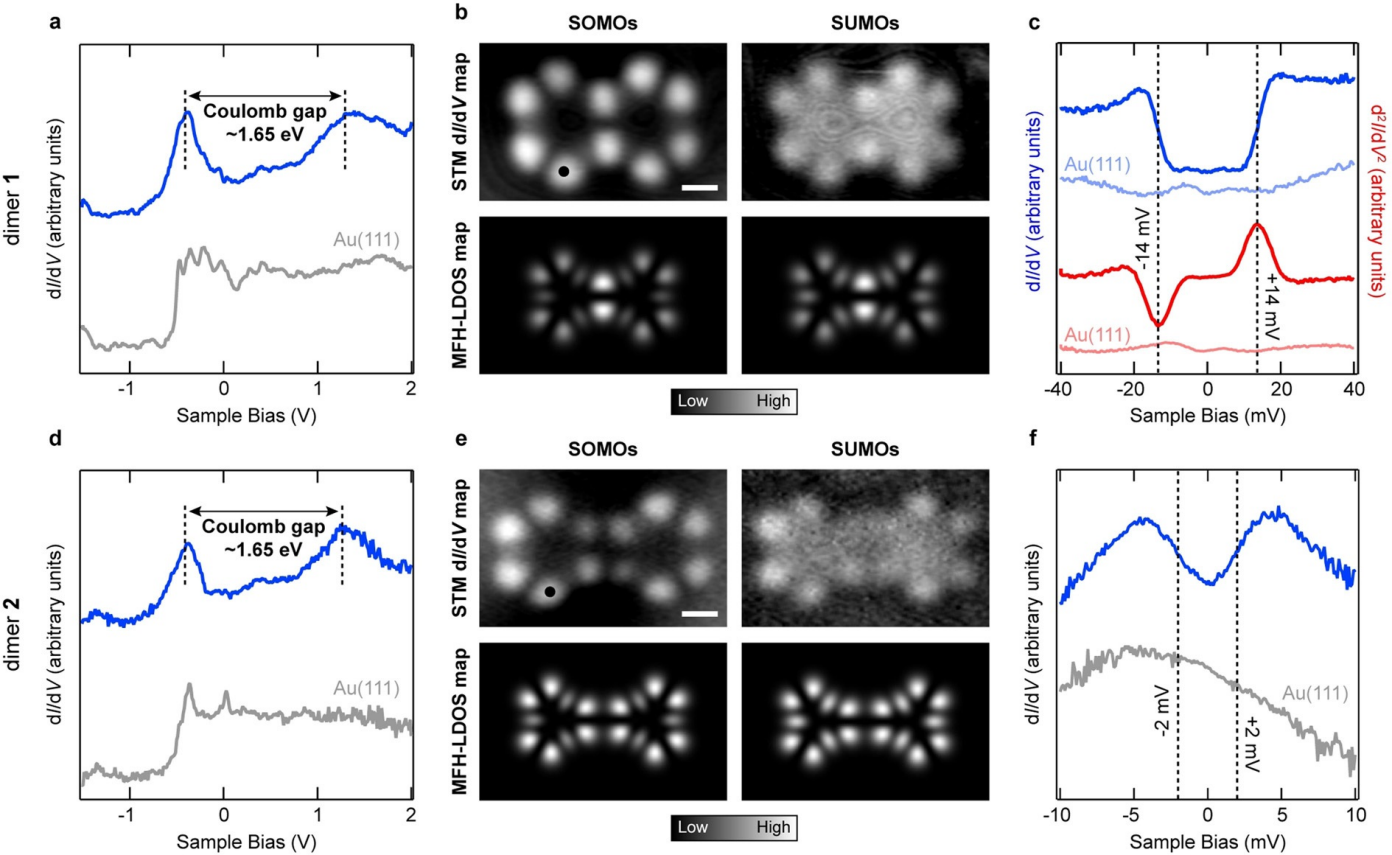
Experimental electronic and magnetic characterization of **1** and **2**. a, d) Long‐range d*I*/d*V* spectrum (blue curves) acquired on **1** (a) and **2** (d). Open feedback parameters: *V*=−1.50 V, *I*=350 pA (a) and *V*=−1.50 V, *I*=400 pA (d); *V*_rms_=16 mV (a) and 10 mV (d). b, e) Experimental d*I*/d*V* maps (top) and MFH‐LDOS maps (bottom) at the SOMO and SUMO resonances of **1** (b) and **2** (e). Tunneling parameters: *V*=−450 mV, *I*=350 pA (SOMOs, b), *V*=+1.00 V, *I*=350 pA (SUMOs, b); *V*=−400 mV, *I*=350 pA (SOMOs, e) and *V*=+1.10 V, *I*=450 pA (SUMOs, e); *V*_rms_=22 mV. c, f) d*I*/d*V* (blue curve) and IETS (red curve) spectra acquired on **1** (c), and d*I*/d*V* spectrum (blue curve) acquired on **2** (f) in the vicinity of the Fermi energy. Acquisition positions for the spectra are indicated by filled circles in Figures 4 b, e. Open feedback parameters: *V*=−40 mV, *I*=500 pA (d*I*/d*V* spectra, c), *V*=−40 mV, *I*=1.2 nA (IETS spectra, c) and *V*=−10 mV, *I*=750 pA (d*I*/d*V* spectra, f); *V*_rms_=400 μV (d*I*/d*V* spectra) and 4 mV (IETS spectra). Scale bars: 0.5 nm.

The predicted outcomes of the theoretical analyses are convincingly demonstrated in our experiments. d*I*/d*V* spectroscopy (where *I* and *V* are current and voltage, respectively) on **1** and **2** revealed broad peaks centered at about −400 mV and +1.25 V (Figures 4 a, d; acquisition positions of the spectra are marked with filled circles in Figures 4 b, e). d*I*/d*V* maps acquired at these biases exhibit close correspondence with the mean‐field Hubbard local density of states (MFH‐LDOS) maps of the SOMOs and SUMOs of **1** and **2** (Figures 4 b, e). This confirms the detection of the spin‐split frontier molecular orbitals of both species, and their Coulomb gaps approximately equal 1.65 eV. d*I*/d*V* spectroscopy on **1** in the vicinity of the Fermi energy revealed conductance steps symmetric around zero bias (Figure 4 c, blue curve; the percentage increase in conductance at the steps with respect to the zero bias conductance *σ*≈35 %), which is indicative of an inelastic excitation.^31^ Given the open‐shell singlet ground state and the open‐shell triplet first excited state of **1**, we ascribe the inelastic excitations to singlet–triplet (*S*=0 to *S*=1) spin excitation, which obeys the IETS spin selection rule that dictates Δ*S*=0, ±1 for magnetic excitations (Supporting Information, Note S2). The excitation threshold was extracted to be ±14 mV from a fit to the experimental IETS spectrum with an antiferromagnetic spin‐1 Heisenberg dimer model,^32^ and provides a direct experimental measure of the *J*_eff_ value (or the singlet–triplet gap) of **1** (Figure 4 c, red curve and Supporting Information, Figure S2). This *J*_eff_ value is comparable to those reported in previous studies of open‐shell nanographenes, such as graphene nanoribbon junctions^24^ (up to 10 meV) or Clar's goblet (23 meV),^25^ which also hosts disjoint ZESs similar to **1**. Similarly, d*I*/d*V* spectroscopy on **2** also presents singlet–triplet spin excitations (Figure 4 f, *σ*≈3 %), albeit with a substantially reduced excitation threshold of ±2 mV, demonstrating the tunability of intertriangulene magnetic coupling. The experimentally observed singlet–triplet gaps of **1** and **2** are in good agreement with CAS(6,6) calculations at reasonable values of *U* (Figure 3 c). We note that inelastic steps may also occur due to vibrational excitations. However, the strong dependence of the excitation threshold on the separation between the triangulene units, and its excellent agreement with the calculated singlet–triplet gaps from multi‐reference methods, is a strong indication of the inelastic excitations in the present case being singlet–triplet spin excitations. Additionally, we also performed STS on fused dimers of **1**, where the triangulene units are separated by a large distance, and hence exhibit negligible overlap of their wave functions (Supporting Information, Figure S3). We consistently observed the lack of any inelastic excitations in the fused dimers, which further strengthens the case for the observed inelastic excitations in the dimers as being spin excitations. Finally, it has been shown in previous reports that the competition between the intramolecular exchange interaction and Kondo screening by metal surfaces in high‐spin nanographenes may lead to an observable Kondo resonance at zero bias.^33^ However, given the maximal overlap of the ZESs in individual triangulene units that exhibit a non‐disjoint character, the intratriangulene ferromagnetic exchange is maximized, which may explain the absence of a Kondo resonance in the dimers, as has also been observed for π‐extended triangulene frameworks on metal surfaces.^11^, ^12^

## Conclusion

In summary, we have demonstrated the on‐surface synthesis of triangulene dimers with and without a 1,4‐phenylene spacer. The magnetic ground states of both dimers are predicted to be the open‐shell singlet, with the first and second excited states being the open‐shell triplet and quintet, respectively. In accordance with theoretical predictions, we experimentally detected singlet–triplet spin excitations, whose strength can be tuned with the spatial separation between the triangulene units. Our results confirm that TZNGs on metal surfaces retain their high‐spin magnetic ground states, and can efficiently couple to give rise to collective magnetism. Given the large exchange interaction of 14 meV and the presumably small magnetic anisotropy in triangulene dimers due to the weak spin–orbit coupling in carbon, our findings should pave the way for the fabrication of magnetic TZNG networks, providing a platform to explore emergent quantum phases and realize technologically relevant magnetic materials.

## Conflict of interest

The authors declare no conflict of interest.

## Supporting information

As a service to our authors and readers, this journal provides supporting information supplied by the authors. Such materials are peer reviewed and may be re‐organized for online delivery, but are not copy‐edited or typeset. Technical support issues arising from supporting information (other than missing files) should be addressed to the authors.

Supplementary
