# Supplementary material for: Collective All‐Carbon Magnetism in Triangulene Dimers
Source: Angew Chem Int Ed Engl. 2020 May 18;59(29):12041–7. doi: 10.1002/anie.202002687 (PMC7383983; doi:10.1002/anie.202002687)
Supplement: Supplementary file 1 — Supplementary [file ANIE-59-12041-s001.pdf]

Angewandte Chemie
Eine Zeitschrift der Gesellschaft Deutscher Chemiker

Supporting Information

Collective All-Carbon Magnetism in Triangulene Dimers**

Shantanu Mishra[+], Doreen Beyer[+], Kristjan Eimre, Ricardo Ortiz, Joaquín Fernández-Rossier, Reinhard Berger, Oliver Gröning, Carlo A. Pignedoli, Roman Fasel, Xinliang Feng, and Pascal Ruffieux**

anie_202002687_sm_miscellaneous_information.pdf

Table of contents:

**1. Materials and methods**	**S2**
**2. Supporting STM, STS and theoretical data**	**S4**
**3. Synthetic procedures**	**S8**
**4. High-resolution mass spectra**	**S13**
**5. NMR characterization**	**S17**
**6. References**	**S24**

1. Materials and methods

1.1 Sample preparation and STM/STS measurements. STM measurements were performed with a Scienta Omicron low-temperature LT-STM operating at 4.5 K and base pressure below 5×10^{-11} mbar. Au(111) single crystal surfaces were prepared by Ar^+ sputtering and annealing cycles. Precursor molecules **3** and **4** were contained in quartz crucibles and deposited at 483 and 530 K, respectively, from a home-built evaporator on Au(111) held at room temperature. STM images and dI/dV maps were acquired in constant-current mode. Unless noted otherwise, gold-coated tungsten tips were used for imaging and spectroscopy. Indicated tunneling biases are provided with respect to the sample. dI/dV and IETS spectra, and dI/dV maps were acquired with a lock-in amplifier operating at a frequency of 860 Hz. Lock-in modulation voltages (root mean square amplitude, V_{rms}) for each measurement is provided in the respective figure captions. The fitting of dI/dV and IETS spectra to extract the spin excitation thresholds were performed using a code developed by Markus Ternes.[1] Ultra-high resolution STM images were acquired with carbon monoxide-functionalized tips, where the molecules are scanned in a constant-height mode, and the current channel is displayed. Open feedback parameters, and subsequent tip approach distances (Δz) for each measurement is provided in the respective figure captions. Carbon monoxide molecules were deposited on Au(111) at a maximum sample temperature of 13 K. The data shown in this study were processed and analyzed with WaveMetrics Igor Pro or WSxM software.[2]

1.2. Tight binding calculations of the electronic structure. The tight-binding calculations of **1** and **2** have been performed by numerically solving the mean-field Hubbard Hamiltonian with nearest neighbor hopping:

$$\hat{H}_{MFH} = -t \sum_{\langle\alpha,\beta\rangle,\sigma} c^{\dagger}_{\alpha,\sigma} c_{\beta,\sigma} + U \sum_{\alpha,\sigma} \langle n_{\alpha,\sigma}\rangle n_{\alpha,\bar{\sigma}} - U \sum_{\alpha} \langle n_{\alpha,\uparrow}\rangle\langle n_{\alpha,\downarrow}\rangle, \tag{S1}$$

Here, $c^{\dagger}_{\alpha,\sigma}$ and $c_{\beta,\sigma}$ denote the spin selective ($\sigma \in \{\uparrow,\downarrow\}$ with $\bar{\sigma} \in \{\downarrow,\uparrow\}$) creation and annihilation operator at neighboring sites α and β, t is the nearest neighbor hopping parameter (with t = 2.7 eV used), U is the on-site Coulomb repulsion, $n_{\alpha,\sigma}$ is the number operator and $\langle n_{\alpha,\sigma}\rangle$ is the mean occupation number at site α. Orbital electron densities, ρ, of the nth-eigenstate with energy E_n have been simulated from the corresponding state vector $a_{n,i,\sigma}$ by:

$$\rho_{n,\sigma}(\vec{r}) = \left|\sum_{i} a_{n,i,\sigma}\phi_{2p_z}(\vec{r}-\vec{r}_i)\right|^2, \tag{S2}$$

where i denotes the atomic site index, and ϕ_{2p_z} denotes the Slater $2p_z$ orbital for carbon.

1.3. Complete Active Space (CAS) calculations. The CAS method, described by Ortiz et al.,[3] can be broken down in the following steps:

1. Solution of the one-orbital tight-binding model for a given structure and choice of hopping parameters. This yields a single particle spectrum and a set of molecular orbitals.
2. Representation of the Hubbard model in the basis of molecular orbitals.
3. Choice of active space orbitals. In our calculations we include the four non-bonding zero energy states and the lowest energy pair of finite energy states above and below the non-bonding states (that is, HOMO−1 and LUMO+1). Inclusion of the HOMO−1 and LUMO+1 orbitals in the active space is necessary, since these states, unlike the zero energy states, are delocalized and extend over both the triangulene units. As shown by Ortiz et al.,[3] inclusion of finite energy states in the active space for systems with disjoint zero energy states is necessary to obtain a finite singlet-triplet splitting.
4. Construction of the many-body configurations for six electrons in six orbitals.

   The number of configurations is $C_6(12) = \binom{12}{6} = 924$.

5. Construction of the many-body matrix Hamiltonian, obtained by the representation of the Hubbard model in this basis.
6. Diagonalization of the many-body matrix and analysis of energy spectrum degeneracies, that permit to identify the multiplets.

1.4. Solution synthesis. Unless otherwise noted, all starting chemical materials were purchased from Sigma Aldrich, TCI, ABCR, and other chemical providers. All starting materials were used as received without further purification. The solution chemical reactions, unless otherwise mentioned, were conducted under air- and moisture-free conditions using a sealed Schlenk system under argon atmosphere, because of handling air- and moisture-sensitive chemical substances. The reaction progress was monitored by thin layer chromatography (TLC), containing silica-coated aluminum plates and fluorescence marker F_{254} (silica 60, F_{254}, Merck). If necessary, crude reaction products were purified by preparative silica gel chromatography (particle size: 40–63 µm, VWR Chemicals) and recycling gel permeation chromatography (*r*GPC). *r*GPC was carried out on JAI HPLC LC 9110 II NEXT instrument with fraction collector FC-3310, and in series connected GPC columns 2H and 1H with chloroform (HPLC grade) as eluent. For structural characterization, proton and carbon nuclear magnetic resonance spectra (^{1}H and ^{13}C-NMR, respectively) were recorded at room temperature (296 K) on a BRUKER AC 300 P NMR instrument, operating at 300 MHz for ^{1}H-NMR and 75 MHz for ^{13}C-NMR. The NMR measurements were carried out in the liquid-state using deuterated dichloromethane as solvent (CD_2Cl_2, 99.8 atom% D, $\delta_{H\text{-}NMR}$ = 5.32 ppm / $\delta_{C\text{-}NMR}$ = 54.2 ppm), purchased from Euroisotop. The peak pattern in ^{1}H-NMR spectra is described by the commonly used abbreviations: s = singlet, d = doublet, t = triplet and m = multiplet. High-resolution matrix-assisted laser desorption/ionization time-of-flight (HR-MALDI-TOF) mass spectra (MS) were obtained in the liquid-state on Autoflex Speed MALDI-TOF instrument from BRUKER, using 1,8-dihydroxyanthrone (dithranol) and *trans*-2-[3-(4-*tert*-Butylphenyl)-2-methyl-2-propenylidene]malononitrile (DCTB) as matrix. High-resolution atmospheric pressure chemical ionization MS (HR-APCI-MS) and electrospray ionization MS (HR-ESI-MS) were recorded with the Agilent 6538 Ultra High Definition Accurate-Mass Q-TOF LC/MC system. Elemental analysis from recrystallized solid compounds was carried out on a varia MICRO cube from Elementar. The solid was burned at 1150 °C for 70 s under oxygen supply. The melting points from solid compounds were determined with the melting point M-560 instrument from BÜCHI. The temperature range was set to 90–340 °C, with a temperature interval of 10 °C/min. The measurements were performed in melting point tubes from Marienfeld (80 x 1.5 mm, one-side open), and the melting point temperature was recorded once the sample was completely melted.

2. Supporting STM, STS and theoretical data

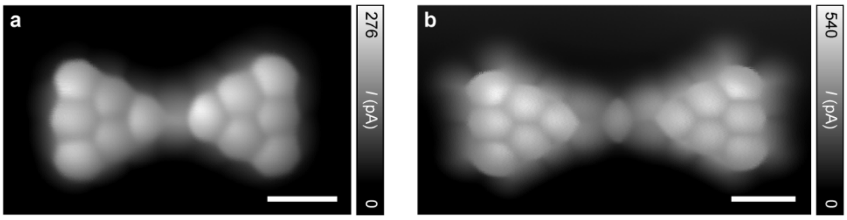

Figure S1. Raw data of ultrahigh-resolution STM images of **1** and **2**. a,b) Ultrahigh-resolution STM images of **1** (a) and **2** (b). The corresponding Laplace-filtered images are shown in Figures 2c,f. Open feedback parameters: $V = –5$ mV, $I = 50$ pA; $\Delta z = –0.8$ Å (a) and –0.9 Å (b). Scale bars: 0.5 nm.

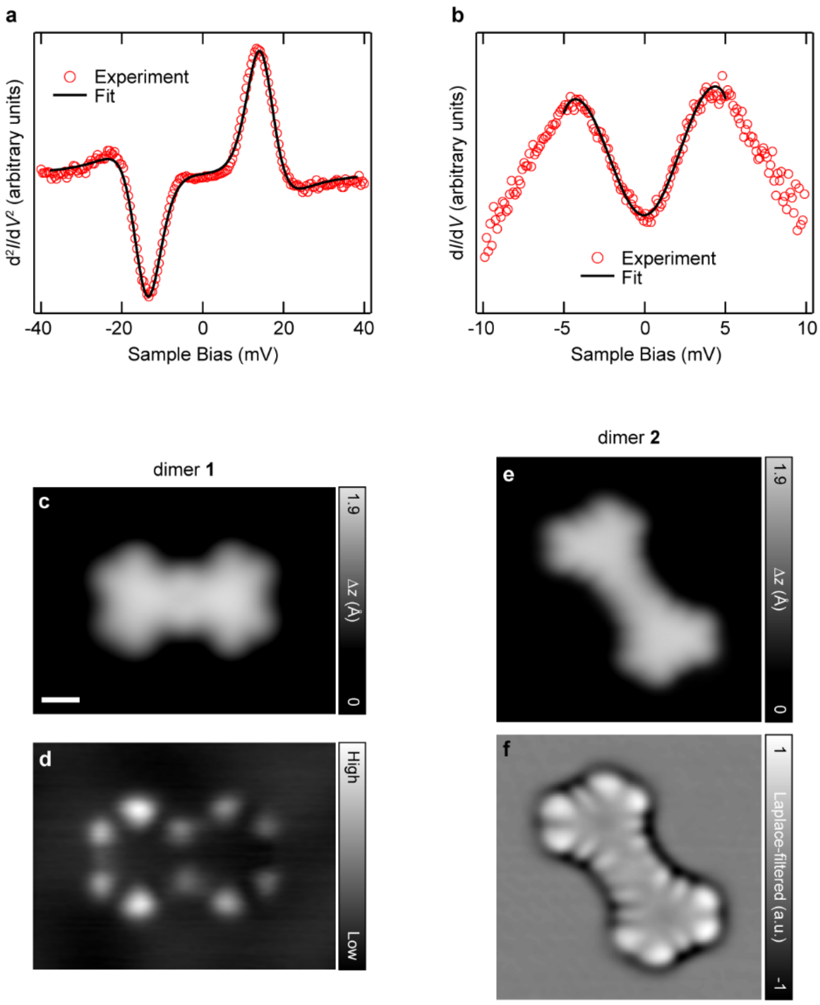

Figure S2. Fits to experimental spin excitation spectra and visualization of the spin excitation signal. a,b) Experimental IETS spectrum acquired on **1** (a) and dI/dV spectrum acquired on **2** (b) in the vicinity of the Fermi energy, revealing singlet-triplet spin excitations (open circles). The data in (a) and (b) are shown in Figures 4c,f. The solid curves are fit to the experimental data, from which spin excitation thresholds of ±14 mV and ±2 mV are extracted for **1** and **2**, respectively. Open feedback parameters: $V = –40$ mV, $I = 1.2$ nA (a) and $V = –10$ mV, $I = 750$ pA (b); $V_{rms} = 4$ mV (a) and 400 µV (b). c,d) High-resolution STM image of **1** (c) and experimental dI/dV map of **1** close to its spin excitation threshold (d). e,f) High-resolution STM image of **2** at its spin excitation threshold (e) and

corresponding Laplace-filtered image revealing the distribution of the spin excitation signal (f). Tunneling parameters: V = –600 mV, I = 200 pA (c), V = –12 mV, I = 180 pA; V_{rms} = 6 mV (d), V = –2 mV, I = 160 pA (e,f).

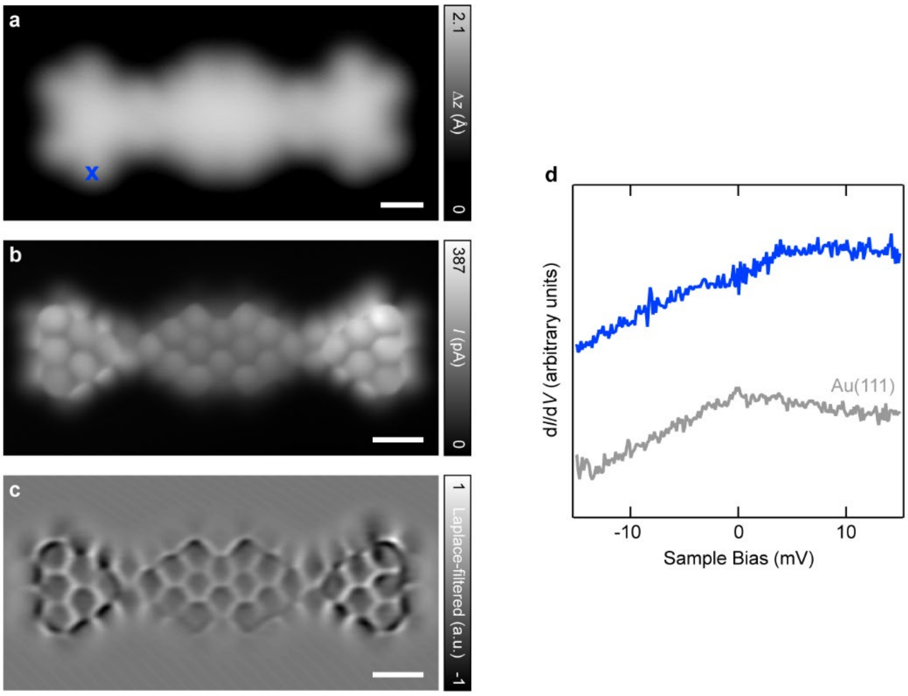

Figure S3. Absence of spin excitations in fused dimers of **1**. a–c) High-resolution (a), ultrahigh-resolution (b) and corresponding Laplace-filtered ultrahigh-resolution (c) STM images of a fused dimer of **1**, where the triangulene units are separated over a large distance. d) dI/dV spectrum acquired on the fused dimer in the vicinity of the Fermi energy (blue curve) revealing absence of inelastic excitations, therefore proving the absence of magnetic coupling between the triangulene units. Acquisition position of the spectrum in (d) is highlighted by a cross in (a). Open feedback parameters: V = –20 mV, I = 500 pA; V_{rms} = 400 µV. Scale bars: 0.5 nm.

Note S1: Solution of the Heisenberg dimer model. The energy spectrum for a Heisenberg dimer Hamiltonian

$$\hat{H} = J\boldsymbol{S_1}.\boldsymbol{S_2}, \tag{S3}$$

where J is the exchange coupling between the spins and $\boldsymbol{S_1}$ and $\boldsymbol{S_2}$ correspond to the individual spin operators, can be obtained using the following trick. We define the total spin operator

$$\boldsymbol{S} = \boldsymbol{S_1} + \boldsymbol{S_2}. \tag{S4}$$

We use the fact that the spectrum of $S^2 = \boldsymbol{S}.\boldsymbol{S}$ is $S(S+1)$, where S are the integer/half integer numbers that cover the range $|S_1 - S_2|, \ldots, S_1 + S_2$. We now write

$$S^2 = (\boldsymbol{S_1} + \boldsymbol{S_2})^2 = S_1^2 + S_2^2 + 2\boldsymbol{S_1}.\boldsymbol{S_2}. \tag{S5}$$

The spectrum of the first two operators on the right hand side of equation (S5) is $S_{1,2}(S_{1,2}+1)$. Therefore, we can write

$$\hat{H} = J\boldsymbol{S_1}.\boldsymbol{S_2} = \frac{J}{2}[S(S+1) - S_1(S_1+1) - S_2(S_2+1)]. \tag{S6}$$

For triangulene, $S_1 = S_2 = 1$, and S can thus take three values, that is, $S = 0$, $S = 1$ and $S = 2$. The energies of the three spin states are given by:

$$E(S) = \frac{J}{2}[S(S+1) - 4]. \tag{S7}$$

For $J > 0$ (that is, antiferromagnetic coupling between the triangulene units), the ground state has $S = 0$, and we have $E(S) - E(0) = (J/2)S(S+1)$, which yields the excitation energies

$$E(1) - E(0) = J \text{ and } E(2) - E(0) = 3J, \tag{S8}$$

as also obtained through the CAS(6,6) method.

Note S2: IETS spin selection rule. Here we elaborate on the origin of the $\Delta S = 0, \pm 1$ spin selection rule for IETS. Our starting point is the assumption that the inelastic co-tunneling event is a spin conserving process when both the molecule and the tunneling electron are considered. Therefore, the initial and final total spin must be conserved, that is

$$S_i(total) = S_f(total). \tag{S9}$$

Now, the initial spin state of the tunneling electron and the molecule is the one obtained from combining the initial spin of the molecule S_i and the $S = 1/2$ for the electron

$$S_i(total) = S_i \pm \frac{1}{2}. \tag{S10}$$

Similarly, the final spin state is also expressed in terms of the final spin of the molecule S_f and the $S = 1/2$ for the electron

$$S_f(total) = S_f \pm \frac{1}{2}. \tag{S11}$$

Now, combining equations (S9)–(S11), we arrive at the condition

$$\Delta S = S_f - S_i = 0, \pm 1. \tag{S12}$$

which provides the selection rule for observing spin excitations in IETS.

3. Synthetic procedures

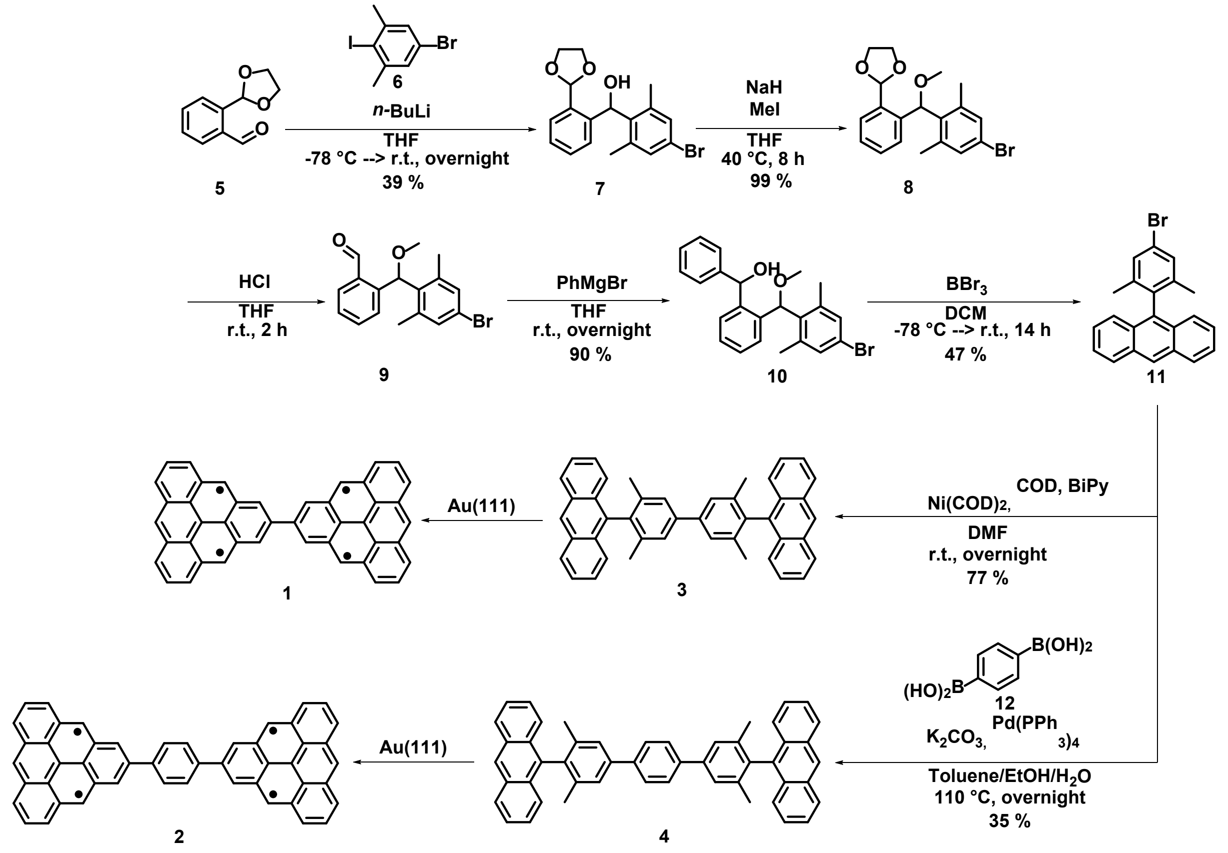

Scheme S1. Summary of synthetic procedures toward formation of **1** and **2**.

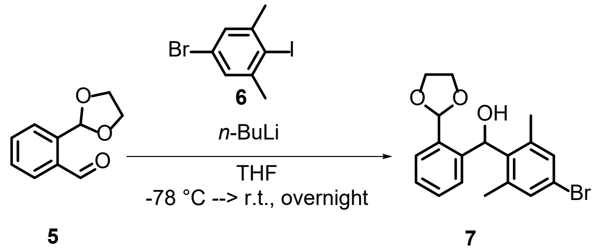

Scheme S2. Synthesis of compound **7**.

(2-(1,3-Dioxolan-2-yl)phenyl(4-bromo-2,6-dimethylphenyl)methanol (7): Commercially available 5-bromo-2-iodo-*m*-xylene (**6**) (3.5 g, 11.2 mmol, 1.0 eq.) was dissolved in 45 ml dry tetrahydrofuran (THF) and cooled to -78 °C. A solution of n-butyl lithium (*n*-BuLi) in hexane (1.6 M, 7.7 ml, 12.3 mmol, 2.2 eq.) was added dropwise under argon atmosphere and the reaction was maintained at -78 °C for 1 hour. Separately, 2-(1,3-dioxolan-2-yl)benzaldehyde (**5**) (1.0 g, 5.6 mmol, 1.0 eq.), synthesized according to literature procedure,[4] was dissolved in 5 ml dry THF and added under argon atmosphere to the reaction mixture via syringe. The resulting mixture was allowed to warm up gradually to room temperature and the reaction mixture stirred until completion. The reaction mixture was quenched with an aqueous solution of ammonium chloride (NH_4Cl), extracted three times with ethyl acetate (EA), and the combined organic layer was washed with brine and dried over magnesium sulfate ($MgSO_4$). The solvent excess was removed by evaporation and the crude compound was purified by silica gel chromatography using EA/*iso*-hexane 1:3 as eluent to afford **7** as white solid (800 mg, 39 %).

^{1}H-NMR (CD_2Cl_2, 300 MHz): δ = 7.60 (dd, J = 7.4, 1.6 Hz, 1H), 7.35 – 7.17 (m, 4H), 6.97 (dd, J = 7.6, 1.4 Hz, 1H), 6.51 (d, J = 3.2 Hz, 1H), 6.03 (s, 1H), 4.28 – 4.03 (m, 4H), 3.72 (d, J = 3.2 Hz, 1H), 2.27 (s, 6H) ppm.

^{13}C-NMR (CD_2Cl_2, 75 MHz): δ = 141.4, 140.4, 137.6, 135. 8, 132.4, 130.2, 128.6, 128.5, 128.5, 121.3, 104.0, 70.3, 66.0, 65.9, 22.0 ppm.

HR-ESI-MS (positive mode): calc. for $[M+Na]^+$: 385.0415, found for $[M+Na]^+$: 385.0401 (deviation: 3.63 ppm).

Melting point: 111.8 °C.

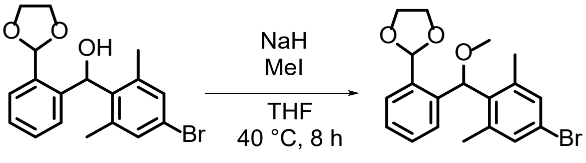

7 **8**

Scheme S3. Synthesis of compound **8**.

2-(2-((4-Bromo-2,6-dimethylphenyl)(methoxy)methyl)phenyl)-1,3-dioxolane (8): A suspension of sodium hydride (NaH) (171.7 mg, 7.1 mmol, 4.0 eq.) in 20 ml dry THF was heated to 40 °C. Compound **7** (650.0 mg, 1.8 mmol, 1.0 eq.), which has been dissolved in 5 ml dry THF, was slowly added under argon atmosphere. The mixture was stirred at 40 °C for 30 minutes. Afterwards, methyl iodide (MeI) (762.0 mg, 5.3 mmol, 3.0 eq.) was added in one portion. After stirring for 8 hours at 40 °C the reaction mixture was cooled to room temperature and the solvent residue was removed under reduced pressure. The crude compound was purified by silica gel chromatography using EA/*iso*-hexane 1:3 as eluent to obtain **8** as sticky light orange oil (670 mg, 99 %).

^{1}H-NMR (CD_2Cl_2, 300 MHz): δ = 7.68 (dd, J = 7.7, 1.4 Hz, 1H), 7.36 – 7.11 (m, 4H), 6.85 (d, J = 7.7 Hz, 1H), 6.15 (s, 1H), 6.12 (s, 1H), 4.22 – 3.96 (m, 4H), 3.31 (d, J = 4.6 Hz, 3H), 2.23 (s, 6H) ppm.

^{13}C-NMR (CD_2Cl_2, 75 MHz): δ = 141.3, 138.5, 138.2, 135.1, 132.3, 129.2, 128. 6, 128.0, 127.0, 121.7, 101.5, 79.8, 65.6, 57.2, 21.1 ppm.

HR-APCI-MS (positive mode): calc. for $[M-OMe]^+$: 345.0485, found for $[M-OMe]^+$: 345.0485.

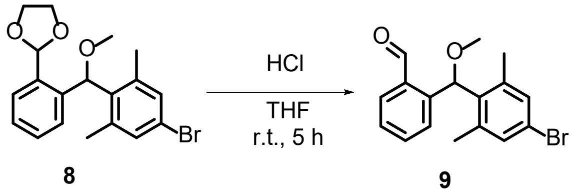

8 **9**

Scheme S4. Synthesis of compound **9**.

2-((4-Bromo-2,6-dimethylphenyl)(methoxy)methyl)benzaldehyde (9): Compound **8** (200.0 mg, 0.5 mmol, 1.0 eq.) was dissolved in a mixture of 6 ml THF and 6 ml 10 % aqueous hydrochloric acid (HCl). The mixture was stirred at room temperature for 5 hours and then neutralized by adding a diluted, aqueous solution of sodium bicarbonate ($NaHCO_3$). After extraction with EA three times, the combined organic phase was washed with brine and dried over $MgSO_4$. The excess of organic solvent was evaporated under reduced pressure and compound **9** was obtained as yellow sticky oil, which has been directly used for the next reaction step without further purification.

^{1}H-NMR (CD_2Cl_2, 300 MHz): δ = 10.36 (s, 1H), 7.95 – 7.81 (m, 1H), 7.42 (pd, J = 7.4, 4.2 Hz, 2H), 7.27 (s, 2H), 6.92 – 6.77 (m, 1H), 6.28 (s, 1H), 3.33 (s, 3H), 2.22 (s, 6H) ppm.

^{13}C-NMR (CD_2Cl_2, 75 MHz): δ = 192.6, 141.6, 141.3, 136.3, 134.2, 133.7, 132.6, 130.3, 129.1, 128.1, 122.2, 79.0, 57.0, 21.2 ppm.

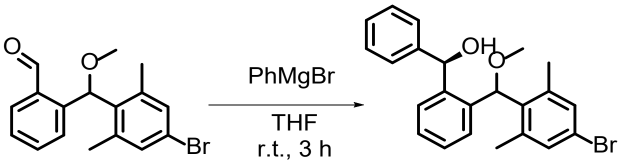

Scheme S5. Synthesis of compound **10**.

(1*R*))-2-((4-Bromo-2,6-dimethylphenyl)(methoxy)methyl)phenyl)(phenyl)metha-nol (10): Compound **9** (500.0 mg, 1.5 mmol, 1.0 eq.) has been dissolved in 5 ml dry THF. A solution of phenylmagnesium bromide (PhMgBr) in hexane (1.0 M, 3.0 ml, 0.54 mmol, 2.0 eq.) was added dropwise and the mixture was stirred at room temperature for 3 hours. After quenching with an aqueous solution of NH_4Cl the mixture was extracted three times with EA, washed with brine and dried over $MgSO_4$. Target compound **10** was isolated as light colorless sticky oil after flash silica gel chromatography using EA/*iso*-hexane 1:3 as eluent (555 mg, 90 %).

^{1}H-NMR (CD_2Cl_2, 300 MHz): δ = 7.41 – 7.24 (m, 11H), 6.71 (d, J = 7.8 Hz, 1H), 6.28 (d, J = 2.8 Hz, 1H), 5.95 (s, 1H), 3.34 (s, 3H), 2.14 (s, 6H) ppm.

^{13}C-NMR (CD_2Cl_2, 75 MHz): δ = 145.5, 143.3, 141.1, 136.7, 134.3, 132.6, 129.3, 129.0, 128.3, 128.1, 128.0, 128.0, 81.0, 72.4, 56.9, 21.1 ppm.

HR-ESI-MS (positive mode): calc. for $[M+Na]^+$: 433.0779, found for $[M+Na]^+$: 433.0769 (deviation: 2.31 ppm).

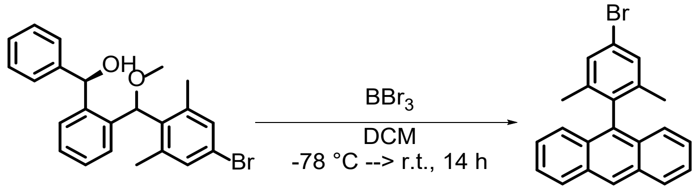

Scheme S6. Synthesis of compound **11**.

9-(4-Bromo-2,6-dimethylphenyl)anthracene (11): Intermediate compound **10** (600.0 mg, 1.5 mmol, 1.0 eq.) was dissolved in 10 ml dry dichloromethane (DCM) and cooled to -78 °C. A solution of boron tribromide (BBr_3) in hexane (1.0 M, 1.75 ml, 1.75 mmol, 1.2 eq.) was added dropwise under argon atmosphere and the solution was allowed to warm up gradually to room temperature. After stirring for 14 hours at room temperature the reaction was quenched with an aqueous solution of NH_4Cl. Afterwards, the mixture was extracted three times with DCM, washed with brine and dried over $MgSO_4$. The crude target was purified by silica gel chromatography using DCM/*iso*-hexane 1:9 as eluent to afford **11** as light yellow solid (250 mg, 47 %).

^{1}H-NMR (CD_2Cl_2, 300 MHz): δ = 8.54 (s, 1H), 8.09 (d, J = 8.5 Hz, 2H), 7.61 – 7.29 (m, 8H), 1.71 (s, 6H) ppm.

^{13}C-NMR (CD_2Cl_2, 75 MHz): δ = 140.7, 137.4, 134.6, 132.2, 130.8, 123.0, 129.3, 127.19, 126.6, 125.9, 125.9, 121.9, 20.1 ppm.

HR-MALDI-TOF (matrix: dithranol): calc. for $[M]^+$: 360.0513, found for $[M]^+$: 360.0565 (deviation: 14.44 ppm).

HR-ESI-MS (positive mode): calc. for $[M]^+$: 360.0513, found for $[M]^+$: 360.0502 (deviation: 3.05 ppm).

Elemental analysis $C_{22}H_{17}Br$: calc. for C: 73.14, H: 4.74; found for C: 73.361, H: 4.576 %.

Melting point: 163.9 °C

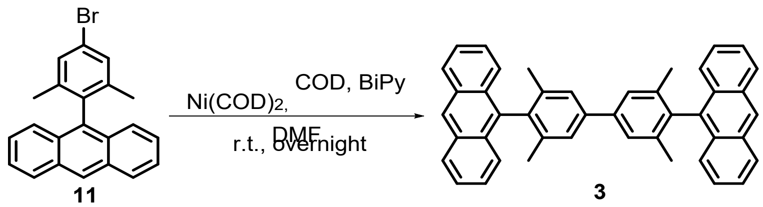

11 3

Scheme S7. Synthesis of compound **3**.

9,9'-(3,3',5,5'-tetramethyl-[1,1'-biphenyl]-4,4'-diyl)dianthracene (3): A mixture of 2,2'-bipyridine (BiPy)) (2.6 mg, 17.0 µmol, 1.2 eq.), bis(1,5-cyclooctadiene)nickel(0) (Ni(COD)$_2$) (4.5 mg, 17 µmol, 1.2 eq.) and 1,5-cyclooctadiene (COD) (1.8 mg, 17 µmol, 11.2 eq.) dissolved in 1 ml *N,N*-dimethylformamide (DMF) has been prepared and stirred at room temperature for 30 minutes under glove box conditions. Afterwards, 9-(4-bromo-2,6-dimethylphenyl)anthracene (**11**) (10.0 mg, 28.0 µmol, 2.0 eq.) was added in one portion and the mixture was stirred at room temperature overnight. Afterwards, the reaction mixture was quenched with water and was extracted three times with DCM. The combined organic layer was washed with brine and dried over $MgSO_4$. After removing the solvent excess under reduced pressure, the crude compound was purified by silica gel chromatography using DCM/*iso*-hexane 1:9 as eluent to afford title compound **3** as light yellow solid (6 mg, 77 %).

^{1}H-NMR (CD_2Cl_2, 300 MHz): δ = 8.56 (s, 2H), 8.12 (d, J = 8.5 Hz, 4H), 7.70 (s, 4H), 7.61 – 7.48 (m, 8H), 7.45 – 7.36 (m, 4H), 1.85 (s, 12H) ppm.

^{13}C-NMR (CD_2Cl_2, 75 MHz): δ = 140.9, 138.8, 137.2, 136.0, 132.3, 130.2, 129.2, 126.8, 126.7, 126.4, 125.9, 20.7.

HR-MALDI-TOF (matrix: DCTB): calc. for $[M]^+$: 562.2660, found for $[M]^+$: 562.2674 (deviation: 2.49 ppm).

HR-APCI-MS (positive mode): calc. for $[M+H]^+$: 563.2739, found for $[M+H]^+$: 563.2728 (deviation: 1.95 ppm).

Elemental analysis $C_{44}H_{34}$: calc. for C: 93.91, H: 6.09; found for C: 93.81, H: 6.13 %.

Melting point: > 340 °C.

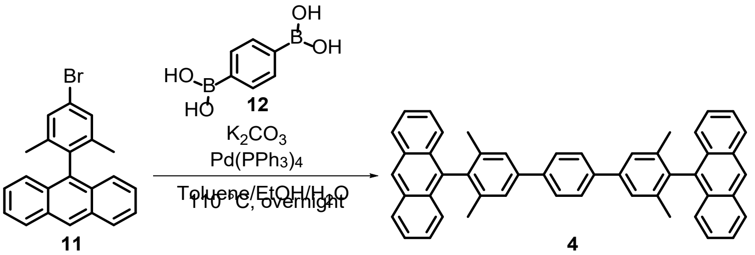

11 4

Scheme S8. Synthesis of compound **4**.

9,9'-(3,3'',5,5''-tetramethyl-[1,1':4',1''-terphenyl]-4,4''-diyl)dianthracene (4): A mixture of 3 ml ethanol (EtOH), 1 ml water (H_2O) and 1 ml toluene has been prepared and intensively purged with argon for at least 15 minutes. Meanwhile, compound **11** (37.0 mg, 0.2 mmol, 1.0 eq.), commercially available 1,4-phenylenediboronic acid (**12**) (201.0 mg, 0.5 mmol, 2.5 eq.) and potassium carbonate (K_2CO_3) (277.0 mg, 2.0 mmol, 9.0 eq.) have been added. At last, tetrakis(triphenylphosphine)palladium(0) ($Pd(PPh_3)_4$) (15.0 mg, 0.01 mmol, 0.06 eq.) was quickly added in one portion and reaction mixture was heated to 110 °C overnight. After cooling down to room temperature the reaction mixture was extracted three times with DCM, washed with brine and dried over $MgSO_4$. The crude material was purified by silica column chromatography using DCM/*iso*-hexane 1:9 as eluent to afford **4** as dark yellow solid (50 mg, 35 %). Further purification by *r*GPC afford **4** as yellow solid.

^{1}H-NMR (CD_2Cl_2, 300 MHz): δ = 8.56 (s, 2H), 8.12 (d, J = 8.5 Hz, 4H), 7.92 (s, 4H), 7.64 (s, 4H), 7.53 (dd, J = 16.0, 8.6 Hz, 8H), 7.44 – 7.36 (m, 4H), 1.84 (s, 12H) ppm.

^{13}C-NMR (CD_2Cl_2, 75 MHz): δ = 140.7, 140.6, 139.1, 137.6, 136.1, 132.5, 130.4, 129.4, 128.2, 127.1, 126.8, 126.6, 126.5, 126.1, 20.71.

HR-MALDI-TOF (matrix: dithranol): calc. for $[M]^+$: 638.2973, found for $[M]^+$: 638.2932 (deviation: 6.4 ppm).

HR-APCI-MS (positive mode): calc. for $[M+H]^+$: 639.3052, found for $[M+H]^+$: 639.3040 (deviation: 1.88 ppm).

Elemental analysis $C_{50}H_{38}$: calc. for C: 94.00, H: 6.00; found for C: 91.95, H: 6.30 %.

Melting point: > 340 °C.

4. High-resolution mass spectra (MALDI-TOF, HR-ESI-MS, HR-APCI-MS)

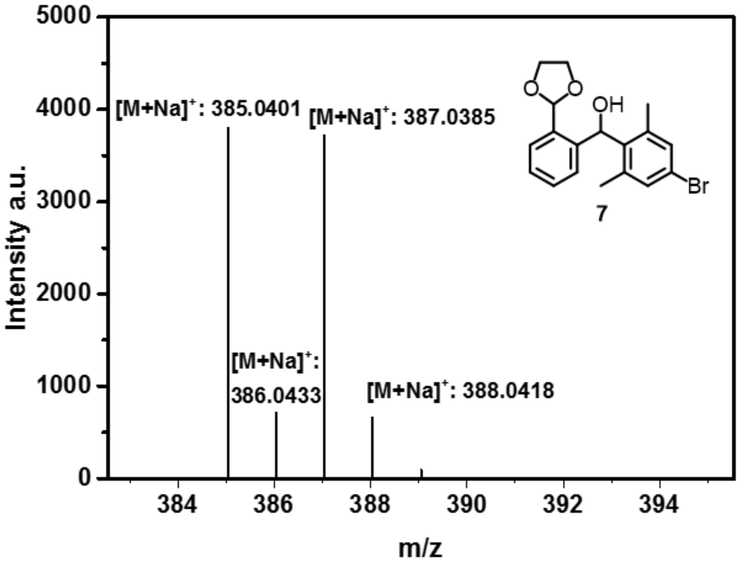

Figure S4. Liquid-state HR-ESI-MS (positive mode) of compound **7**.

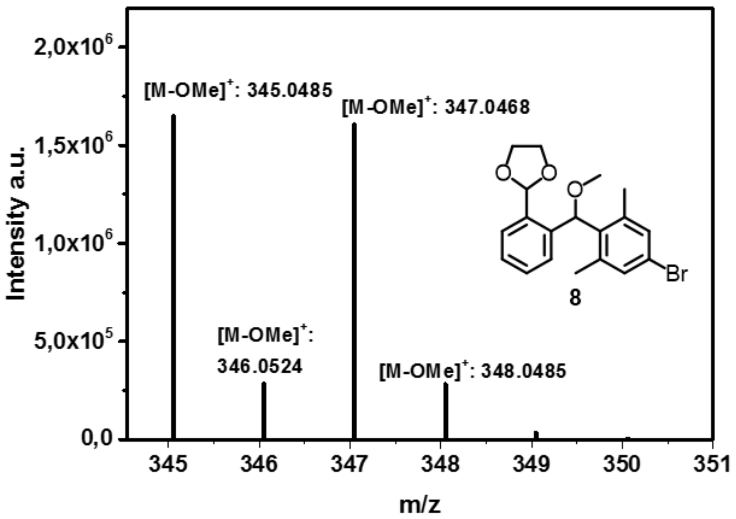

Figure S5. Liquid-state HR-APCI-MS (positive mode) of compound **8**.

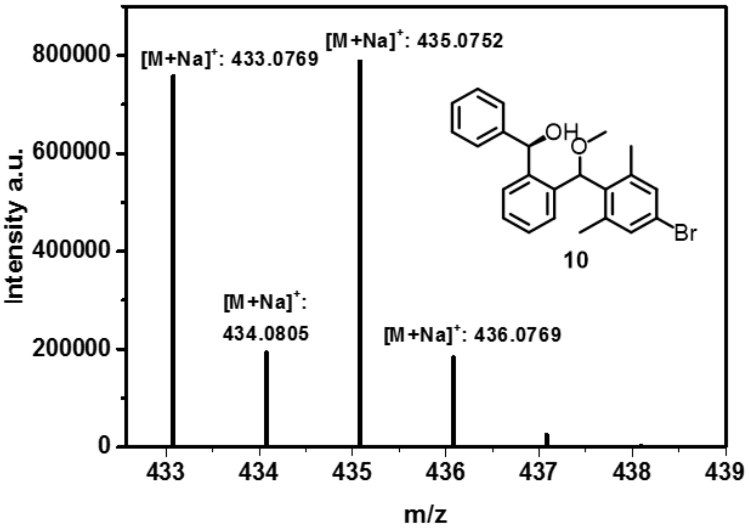

Figure S6. Liquid-state HR-ESI-MS (positive mode) of compound **10**.

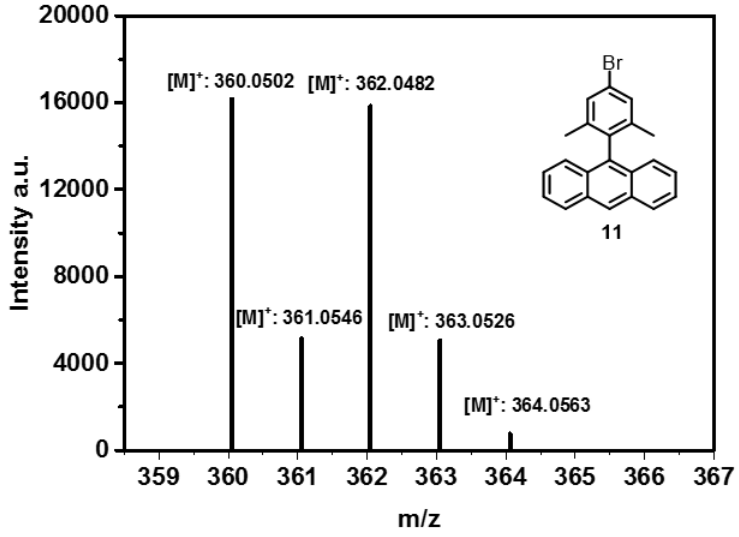

Figure S7. Liquid-state HR-ESI-MS (positive mode) of compound **11**.

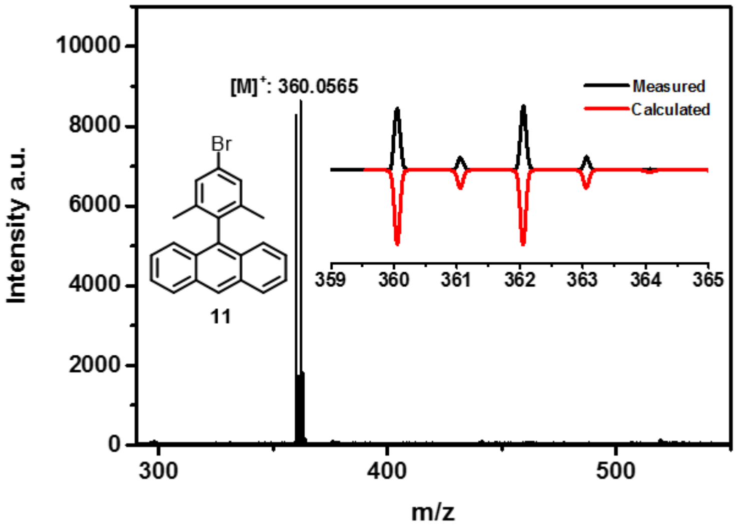

Figure S8. Liquid-state HR-MALDI-TOF (positive mode) of compound **11** (matrix: dithranol).

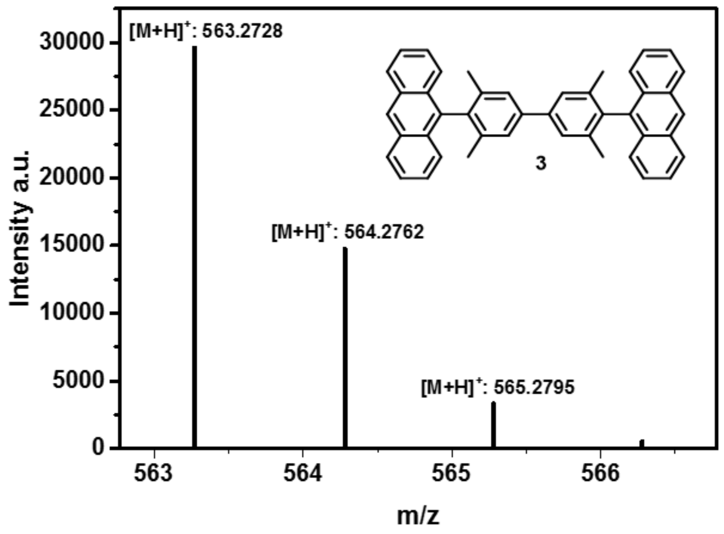

Figure S9. Liquid-state HR-APCI-MS (positive mode) of compound **3**.

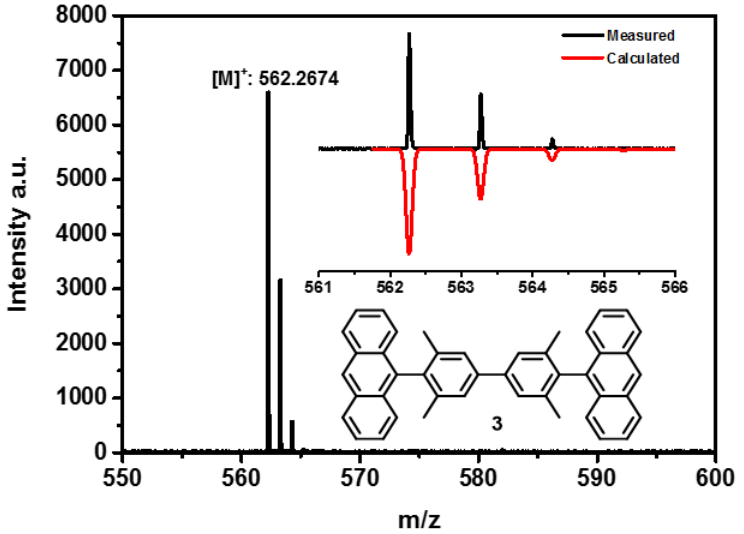

Figure S10. Liquid-state HR-MALDI-TOF (positive mode) of compound **3** (matrix: DCTB).

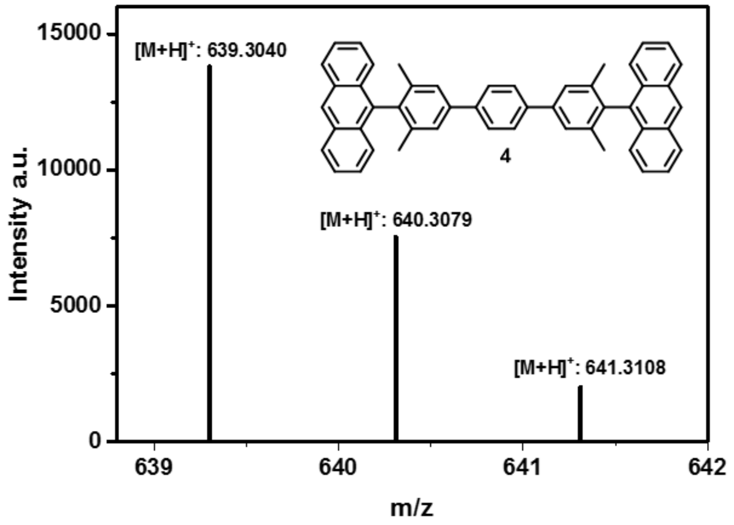

Figure S11. Liquid-state HR-APCI-MS (positive mode) of compound **4**.

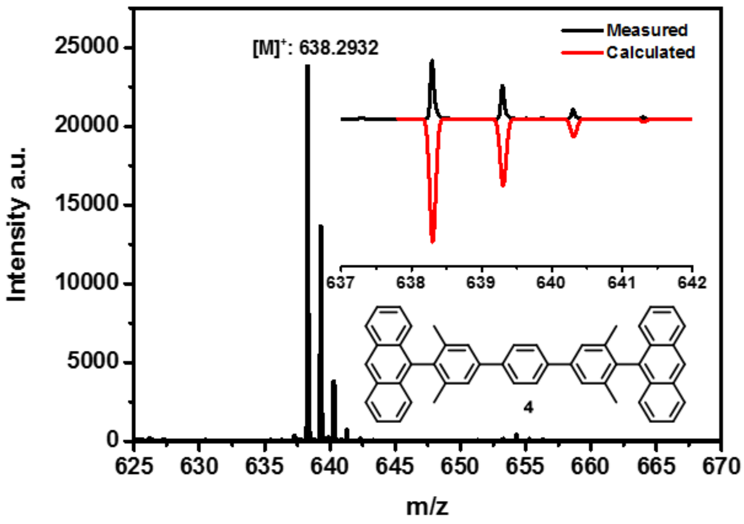

Figure S12. Liquid-state HR-MALDI-TOF (positive mode) of compound **4** (matrix: dithranol).

5. NMR Characterization (^{1}H-, ^{13}C- and 2D-NMR)

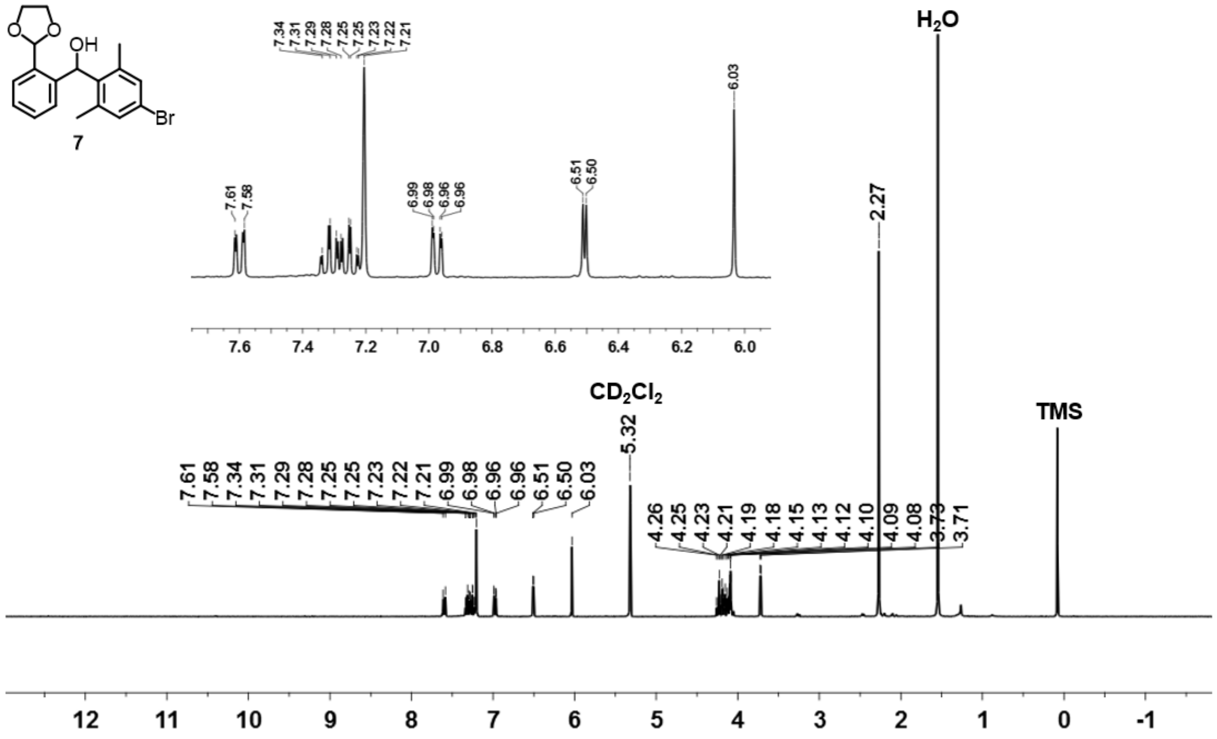

Figure S13. Liquid-state ^{1}H-NMR spectrum of compound **7** measured in CD_2Cl_2 at room temperature. Frequency: 300 MHz.

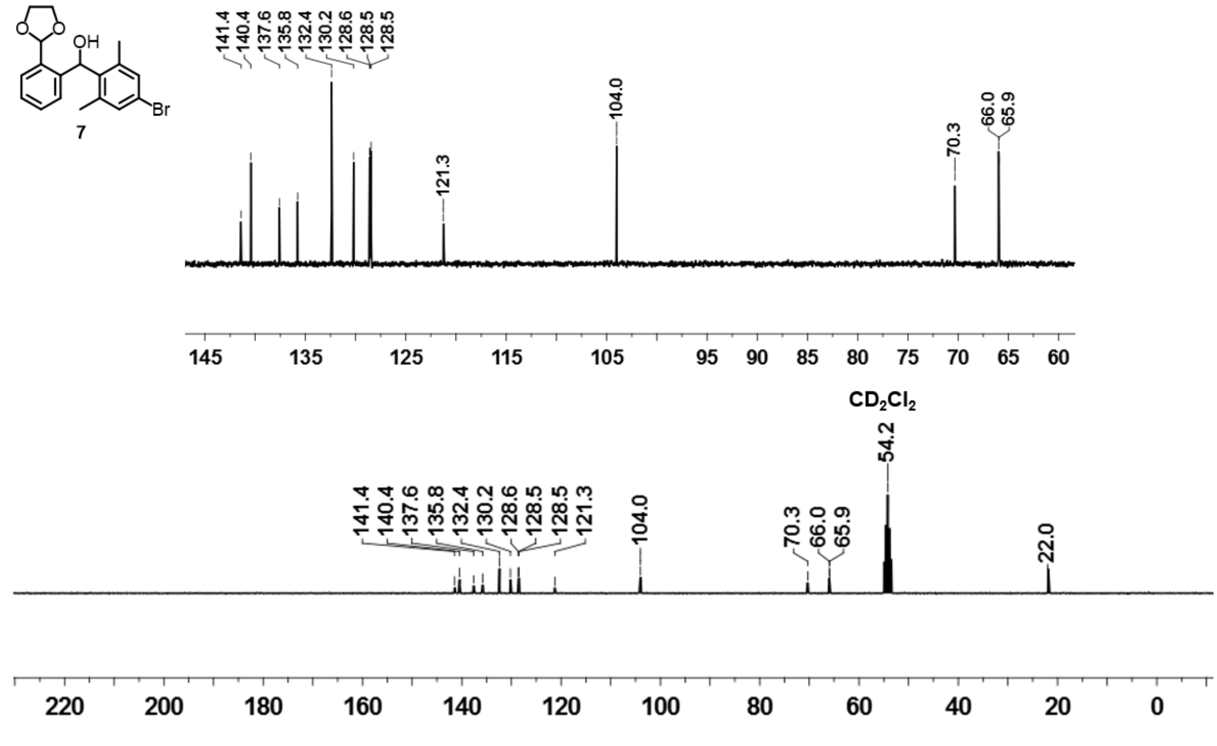

Figure S14. Liquid-state ^{13}C-NMR spectrum of compound **7** measured in CD_2Cl_2 at room temperature. Frequency: 75 MHz.

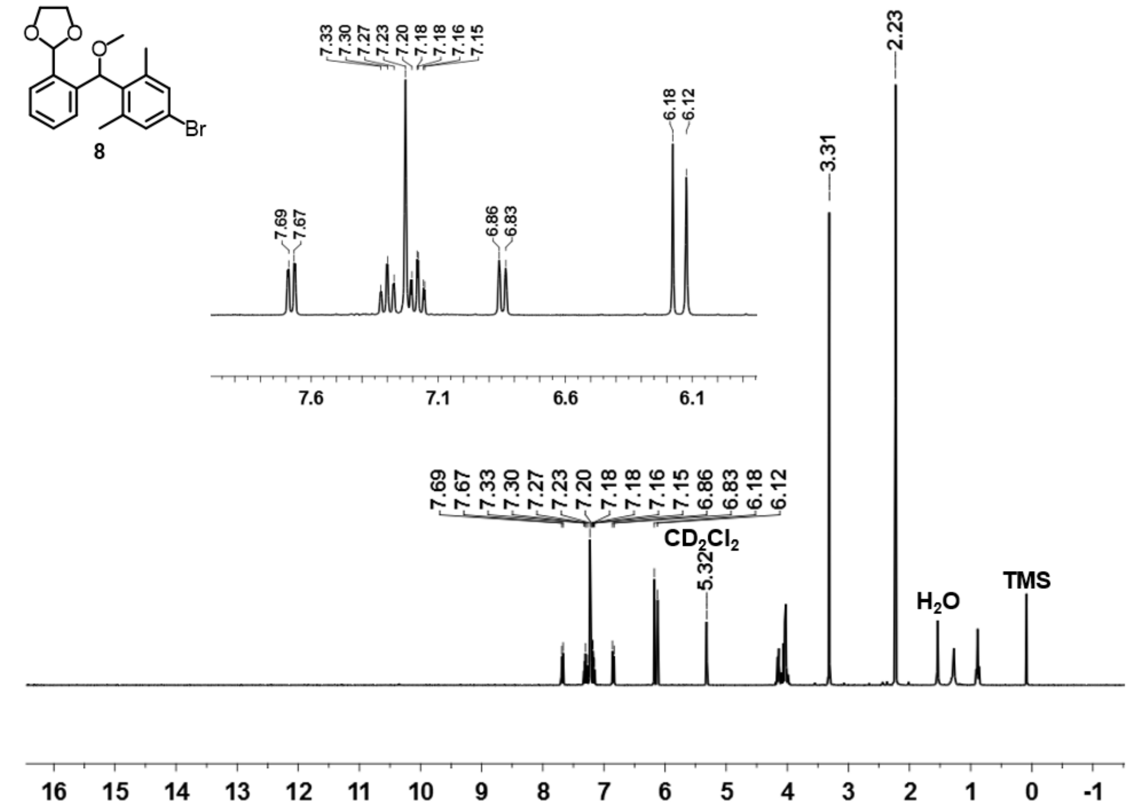

Figure S15. Liquid-state ^{1}H-NMR spectrum of compound **8** measured in CD_2Cl_2 at room temperature. Frequency: 300 MHz.

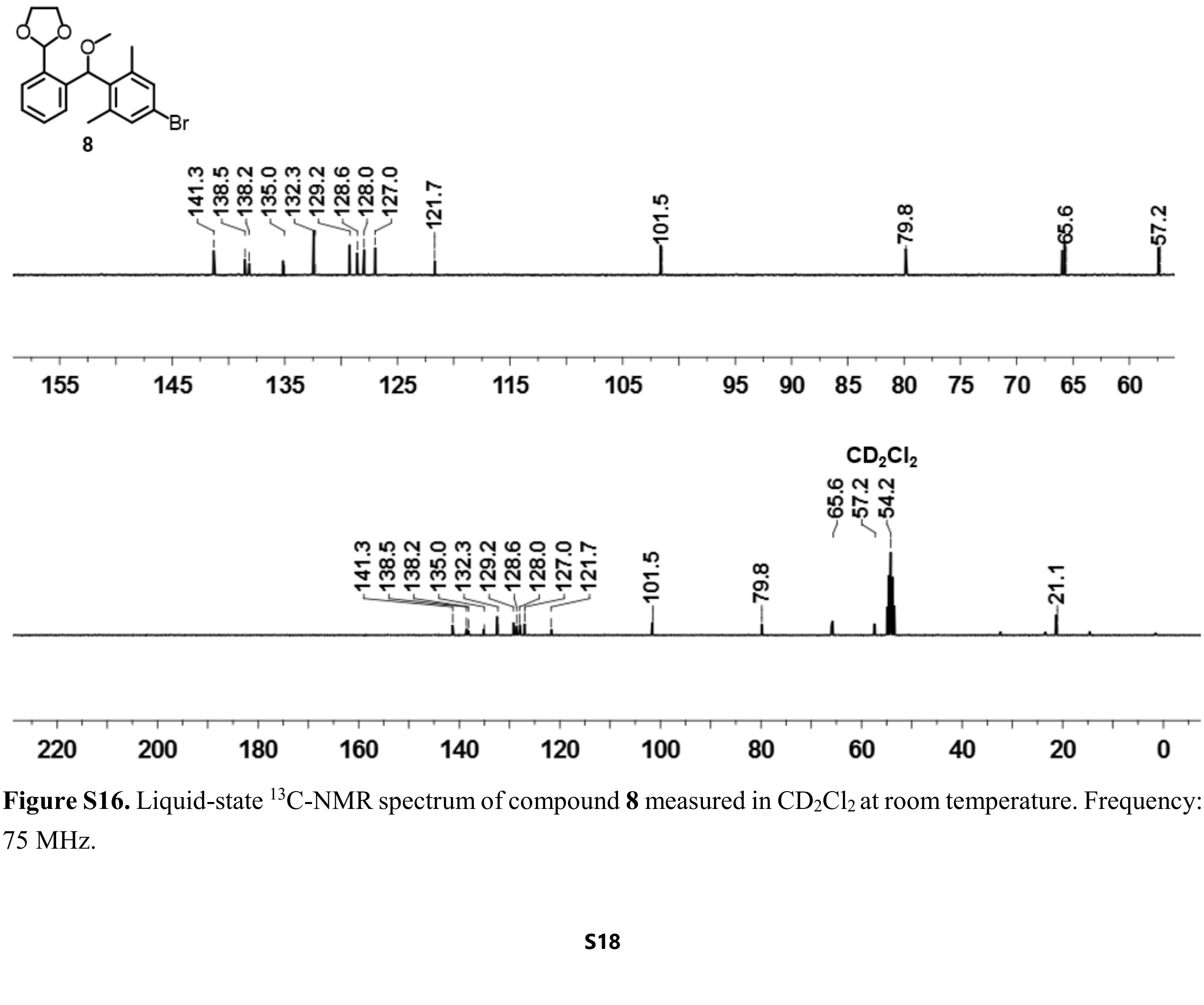

Figure S16. Liquid-state ^{13}C-NMR spectrum of compound **8** measured in CD_2Cl_2 at room temperature. Frequency: 75 MHz.

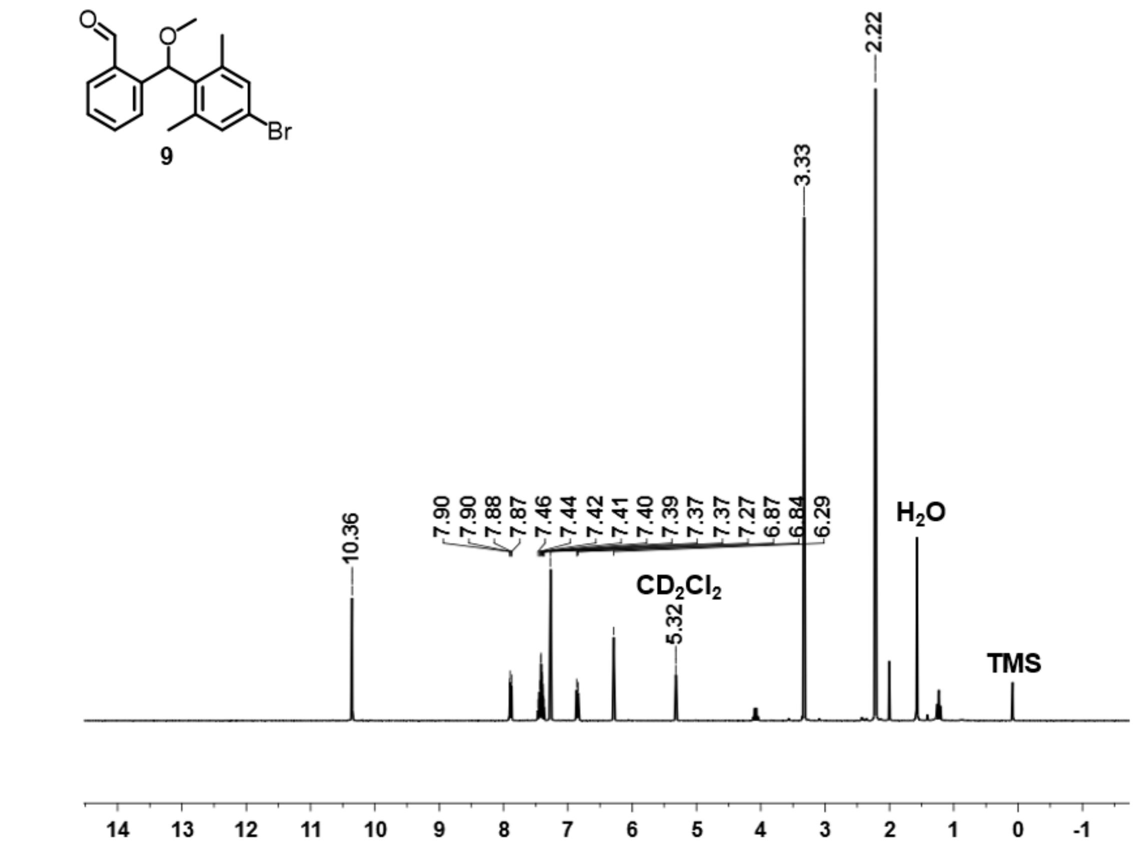

Figure S17. Liquid-state ^{1}H-NMR spectrum of compound **9** measured in CD_2Cl_2 at room temperature. Frequency: 300 MHz.

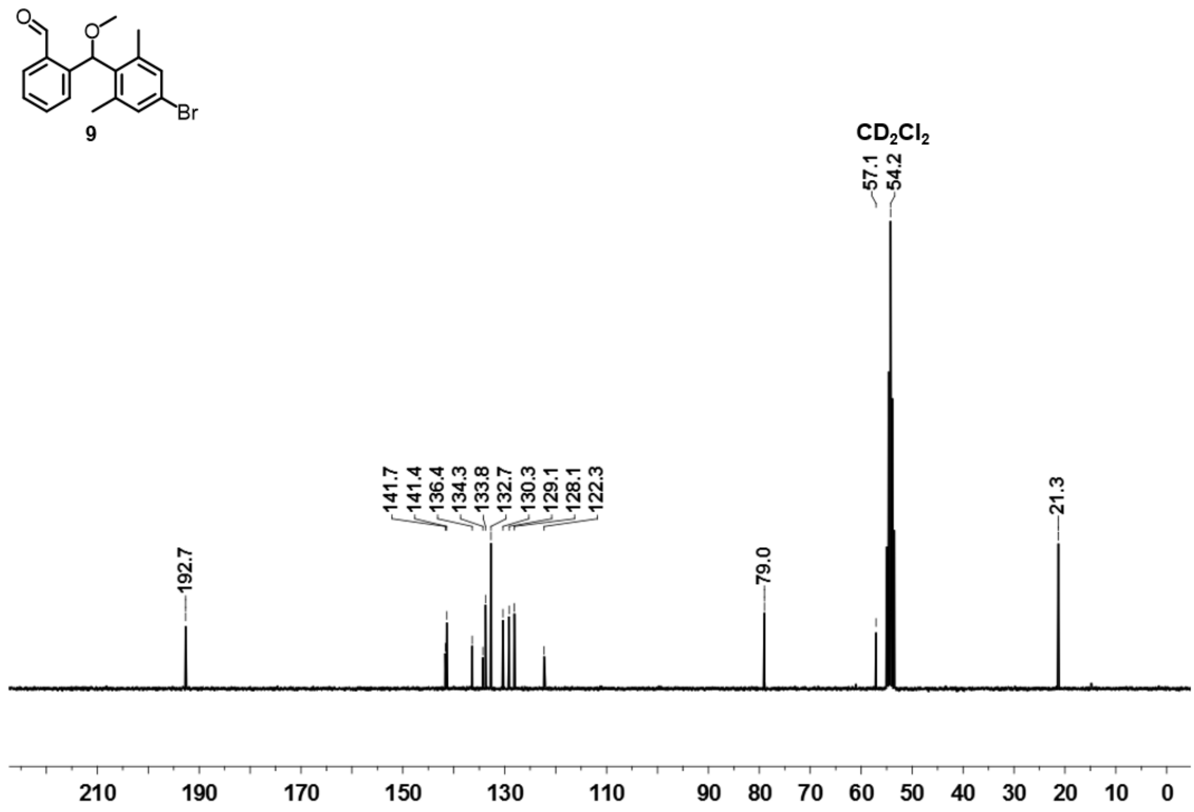

Figure S18. Liquid-state ^{13}C-NMR spectrum of compound **9** measured in CD_2Cl_2 at room temperature. Frequency: 75 MHz.

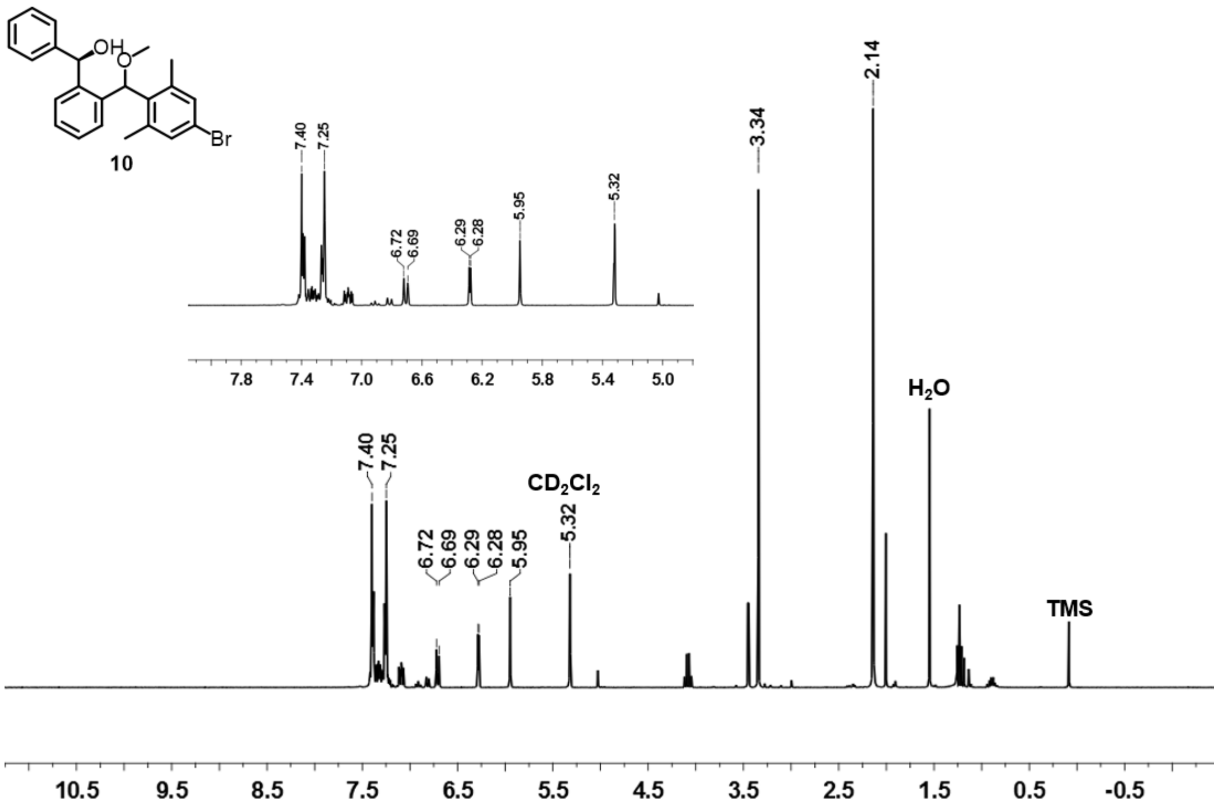

Figure S19. Liquid-state ^{1}H-NMR spectrum of compound **10** measured in CD_2Cl_2 at room temperature. Frequency: 300 MHz.

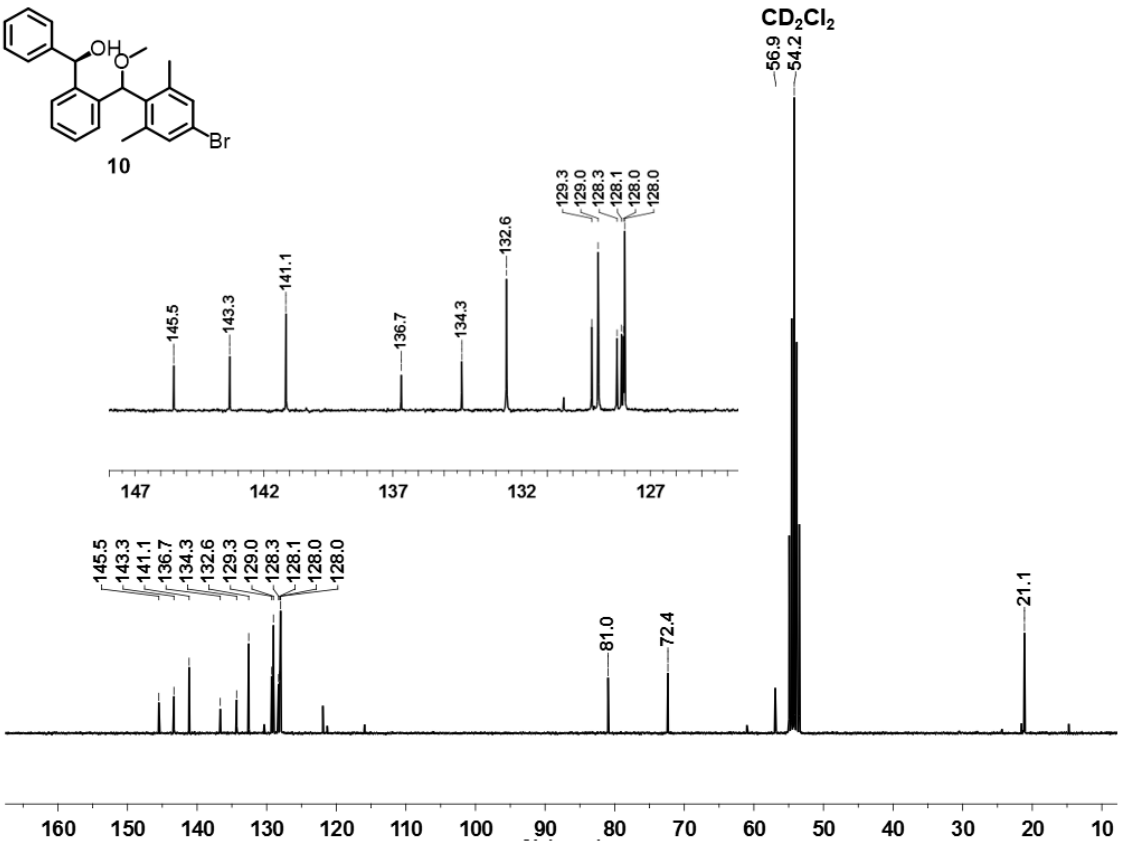

Figure S20. Liquid-state ^{13}C-NMR spectrum of compound **10** measured in CD_2Cl_2 at room temperature. Frequency: 75 MHz.

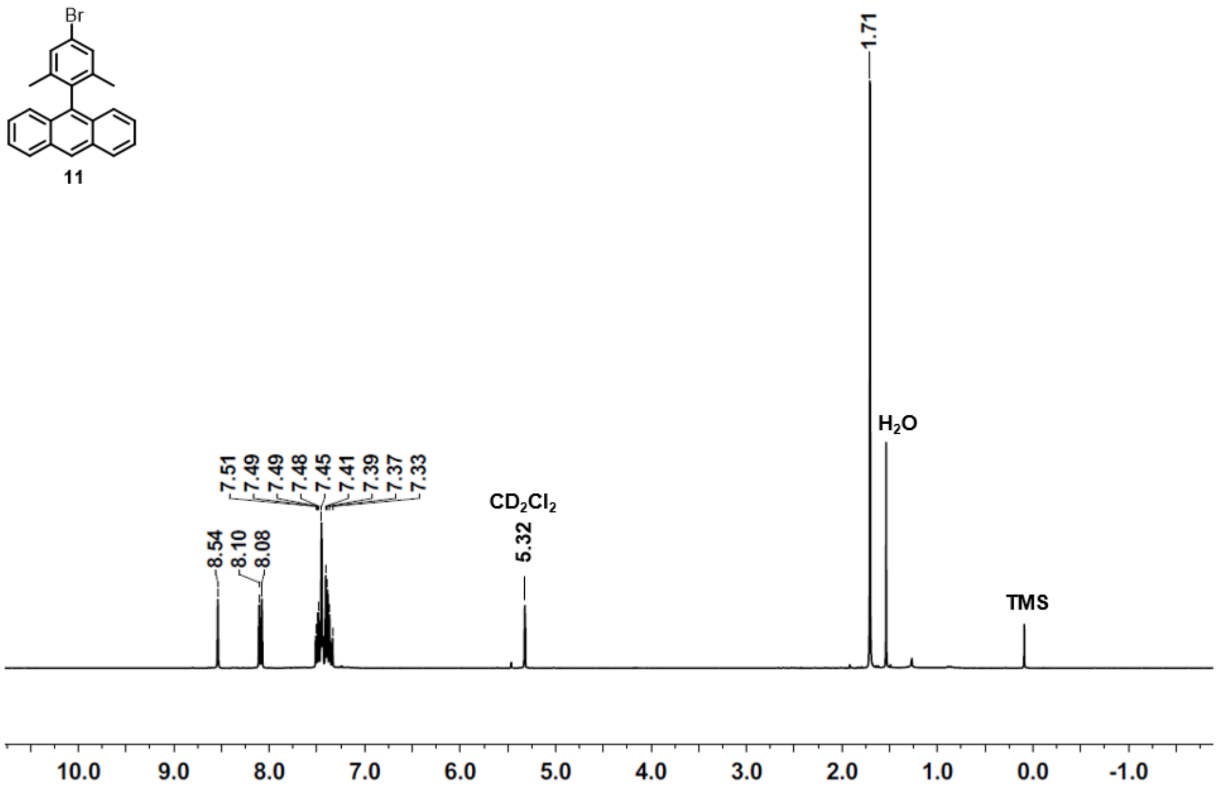

Figure S21. Liquid-state ^{1}H-NMR spectrum of compound **11** measured in CD_2Cl_2 at room temperature. Frequency: 300 MHz.

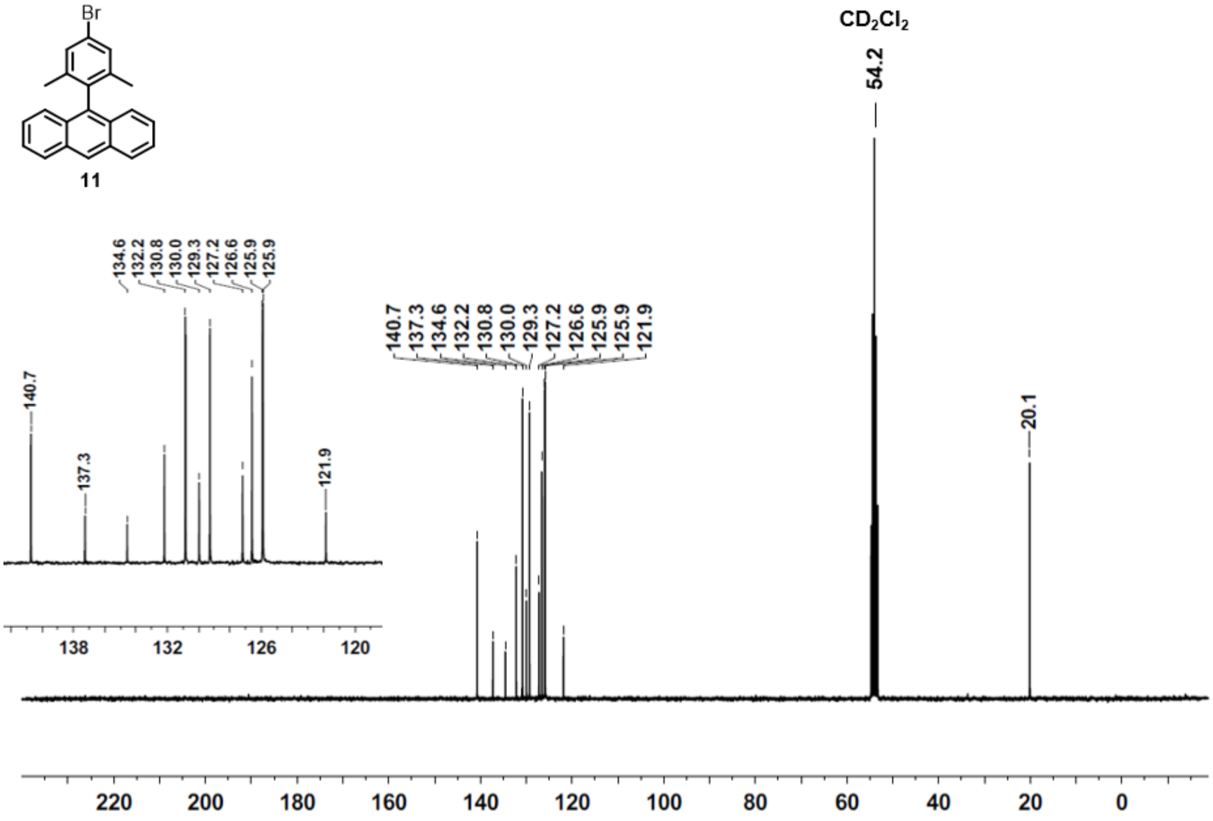

Figure S22. Liquid-state ^{13}C-NMR spectrum of compound **11** measured in CD_2Cl_2 at room temperature. Frequency: 75 MHz.

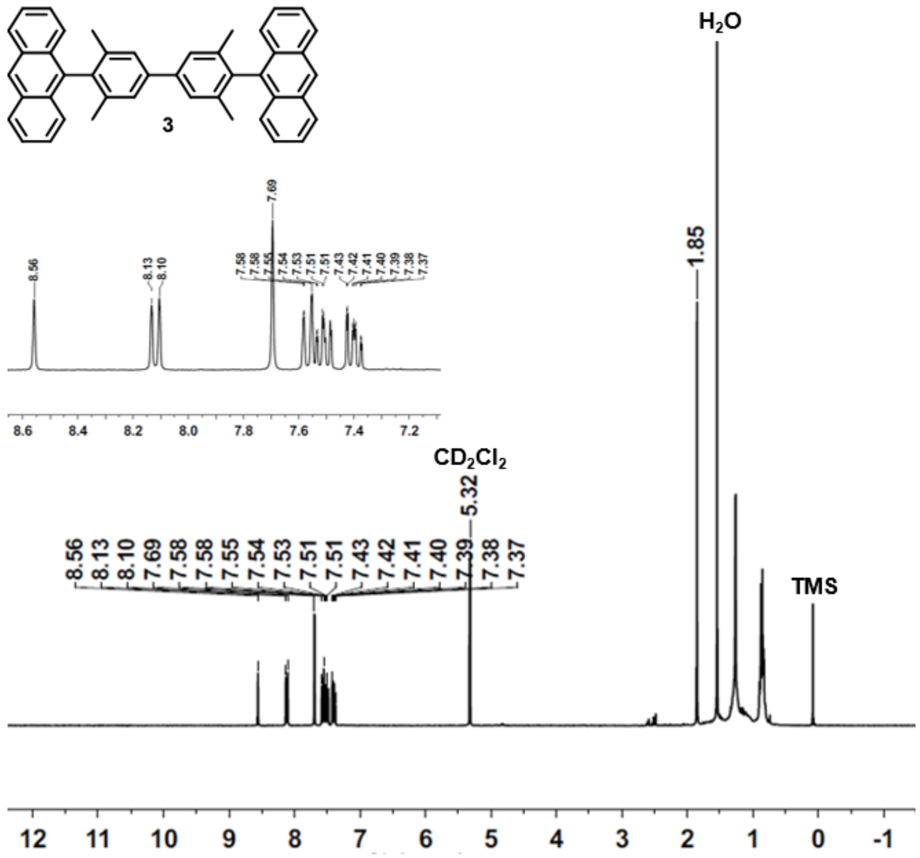

Figure S23. Liquid-state ^{1}H-NMR spectrum of compound **3** measured in CD_2Cl_2 at room temperature. Frequency: 300 MHz.

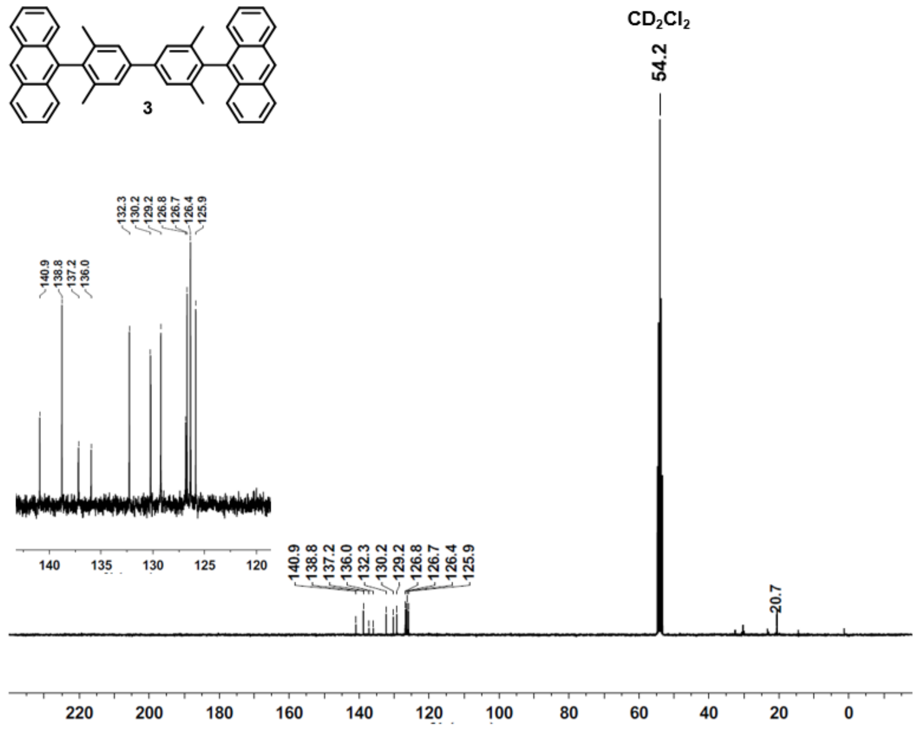

Figure S24. Liquid-state ^{13}C-NMR spectrum of compound **3** measured in CD_2Cl_2 at room temperature. Frequency: 75 MHz.

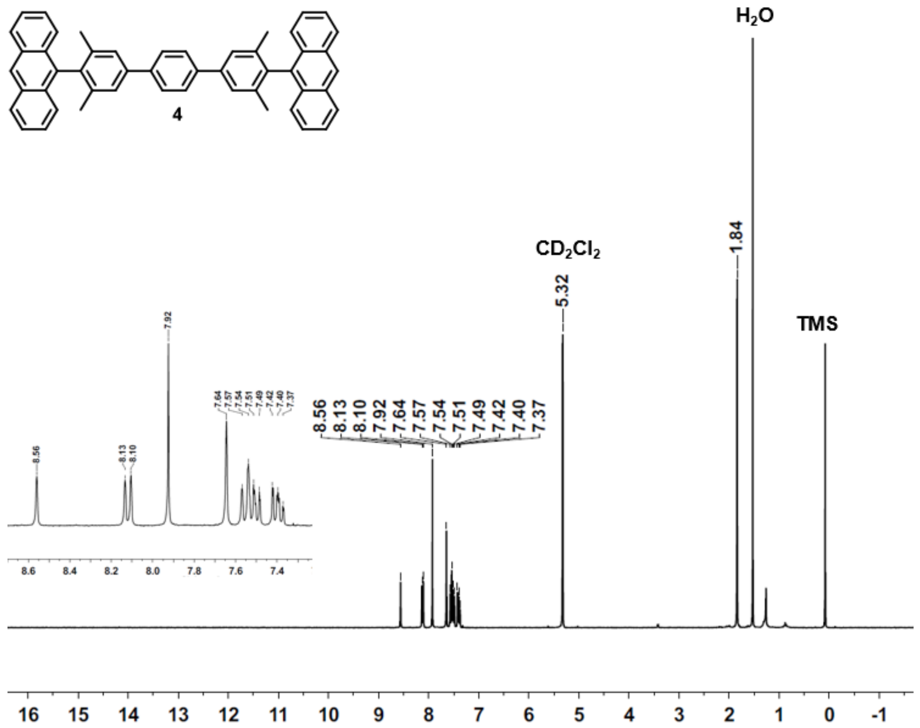

Figure S25. Liquid-state ^{1}H-NMR spectrum of compound **4** measured in CD_2Cl_2 at room temperature. Frequency: 300 MHz.

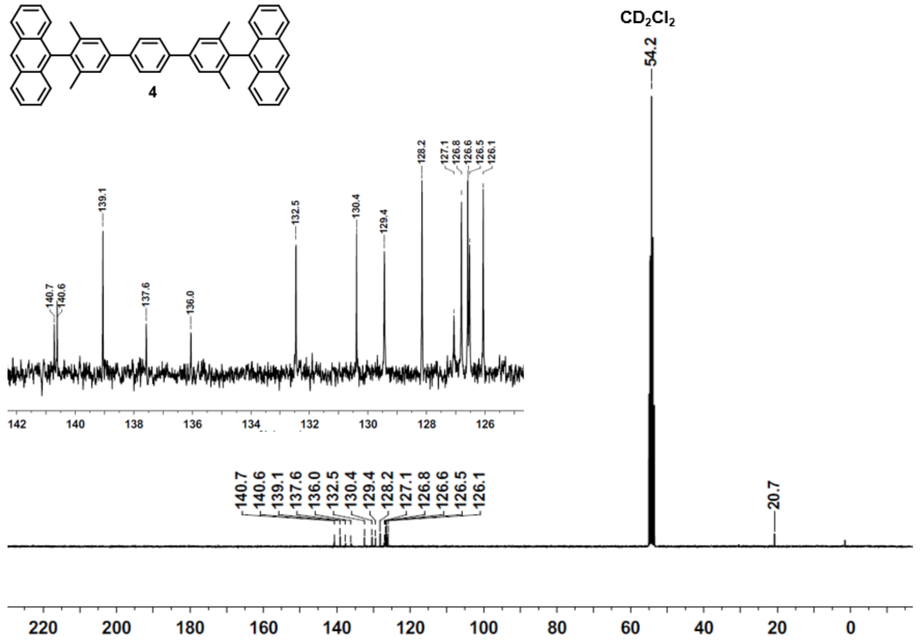

Figure S26. Liquid-state ^{13}C-NMR spectrum of compound **4** measured in CD_2Cl_2 at room temperature. Frequency: 75 MHz.

6. References

[1] M. Ternes, *New J. Phys.* **2015**, *17*, 063016.

[2] I. Horcas, R. Fernández, J. M. Gómez-Rodríguez, J. Colchero, J. Gómez-Herrero, A. M. Baro, *Review of Scientific Instruments* **2007**, *78*, 013705.

[3] R. Ortiz, R. A. Boto, N. García-Martínez, J. C. Sancho-García, M. Melle-Franco, J. Fernández-Rossier, *Nano Lett.* **2019**, *19*, 5991–5997.

[4] R. Liang, T. Ma, S. Zhu, *Org. Lett.* **2014**, *16*, 4412–4415.
